# Genetic and species rearrangements in microbial consortia impact biodegradation potential

**DOI:** 10.1093/ismejo/wraf014

**Published:** 2025-01-25

**Authors:** Zaki Saati-Santamaría, Pilar Navarro-Gómez, Juan A Martínez-Mancebo, Maitane Juárez-Mugarza, Amando Flores, Inés Canosa

**Affiliations:** Departamento de Microbiología y Genética, Universidad de Salamanca, 37007 Salamanca, Spain; Institute for Agrobiotechnology Research (CIALE), Universidad de Salamanca, 37185 Salamanca, Spain; Laboratory of Fungal Genetics and Metabolism, Institute of Microbiology of the Czech Academy of Sciences, 14200 Prague, Czech Republic; Department of Molecular Biology and Biochemistry, Universidad Pablo de Olavide, Centro Andaluz de Biología del Desarrollo/Consejo Superior de Investigaciones Científicas/Junta de Andalucía, 41013 Seville, Spain; Department of Molecular Biology and Biochemistry, Universidad Pablo de Olavide, Centro Andaluz de Biología del Desarrollo/Consejo Superior de Investigaciones Científicas/Junta de Andalucía, 41013 Seville, Spain; Department of Molecular Biology and Biochemistry, Universidad Pablo de Olavide, Centro Andaluz de Biología del Desarrollo/Consejo Superior de Investigaciones Científicas/Junta de Andalucía, 41013 Seville, Spain; Department of Plant Biology and Ecology, Faculty of Science and Technology, The University of the Basque Country (UPV/EHU), Barrio Sarriena s/n, 48940 Leioa, Spain; Department of Molecular Biology and Biochemistry, Universidad Pablo de Olavide, Centro Andaluz de Biología del Desarrollo/Consejo Superior de Investigaciones Científicas/Junta de Andalucía, 41013 Seville, Spain; Department of Molecular Biology and Biochemistry, Universidad Pablo de Olavide, Centro Andaluz de Biología del Desarrollo/Consejo Superior de Investigaciones Científicas/Junta de Andalucía, 41013 Seville, Spain

**Keywords:** ibuprofen, microbial ecology, consortia evolution, biodegradation, emerging pollutants

## Abstract

Genomic reorganisation between species and horizontal gene transfer have been considered the most important mechanism of biological adaptation under selective pressure. Still, the impact of mobile genes in microbial ecology is far from being completely understood. Here we present the collection and characterisation of microbial consortia enriched from environments contaminated with emerging pollutants, such as non-steroidal anti-inflammatory drugs. We have obtained and further enriched two ibuprofen-degrading microbial consortia from two unrelated wastewater treatment plants. We have also studied their ability to degrade the drug and the dynamics of the re-organisations of the genetic information responsible for its biodegradation among the species within the consortium. Our results show that genomic reorganisation within microorganisms and species rearrangements occur rapidly and efficiently during the selection process, which may be facilitated by plasmids and/or transposable elements located within the sequences. We show the evolution of at least two different plasmid backbones on samples from different locations, showing rearrangements of genomic information, including genes encoding activities for IBU degradation. As a result, we found variations in the expression pattern of the consortia after evolution under selective pressure, as an adaptation process to the new conditions. This work provides evidence for changes in the metagenomes of microbial communities that allow adaptation under a selective constraint –ibuprofen as a sole carbon source– and represents a step forward in knowledge that can inspire future biotechnological developments for drug bioremediation.

## Introduction

Microorganisms are known to play a key role in most biogeochemical cycles on Earth [1]. Bacteria, in particular, can metabolise a wide range of common pollutants thanks to their broad metabolic capabilities and the evolutionary capacity of their genes [2–4]. These compounds, that include synthetic chemicals that are foreign to the organisms of an ecological system, also known as xenobiotics, may act as a selective pressure that may force adaptation which can be mediated by single nucleotide polymorphisms (SNPs), gene duplication or loss, and by more complex processes involving the horizontal gene transfer (HGT) of large regions of DNA [5, 6]. HGT accelerates bacterial adaptation by introducing new functionalities into microbiomes that are transferred by mobile genetic elements (e.g.: plasmids, transposases, bacteriophages) [6, 7]. As a result, gene mobilisation generates new variants that are responsible of the major genetic diversity within species [8]. These variants can modify, not only strains but entire microbial communities [6, 9–11]. Overall, HGT events provide a more complex scenario for fully understanding the dynamics, success and adaptation of microbial communities [12, 13]. Catabolic plasmids bear the genes encoding the enzymes involved in the biodegradation of xenobiotic compounds and are commonly of low copy number, large size (50–500 kbp) and self-transmissible [14]. Biodegradation genes are frequently encoded by transposons or flanked by insertion sequences (ISs) that may have been involved in the recruitment of these genes by the plasmids and their subsequent dispersal among other replicons and hosts [15].

Bioremediation processes take advantage of the metabolic capabilities of microorganisms to degrade complex contaminants and, combined with the extensive genome information stored in databases that facilitates discovering new biodegradative genes, have emerged as innovative and promising solutions. Bacterial species have been identified as having the potential to fully or partially degrade emerging pollutants, and specifically pharmaceuticals that are increasingly produced and consumed during the last few decades [16–18]. Among these, non-steroidal anti-inflammatory drugs have gained significant attention due to their persistence and potential adverse effects on environment and human health. In particular, ibuprofen (IBU) exhibits high stability and biological activity at low concentrations and a prolonged release to the environment over time, which implies a high risk of long-term exposure, leading to large adverse effects on aquatic and terrestrial organisms [19–21].

To date, some bacterial strains are known to use IBU as a sole source of carbon and energy, among them, *Sphingomonas* sp. Ibu-2 [22], *Rhizorhabdus wittichii* MPO218 (formerly *Sphingomonas wittichii*) [23] and *Sphingopyxis granuli* RW412 [24]. All of them belong to the *Sphingomonadaceae* family and contain a group of genes named *ipf*, which encode proteins responsible for the IBU catabolic pathway. These genes are arranged in different genomic organisations, and they participate in the so-called upper pathway, which catalyses the transformation of IBU into its catechol derivative, and a lower pathway responsible for the transformation of this compound into the tricarboxylic acid intermediates [24–26]. The upper pathway is mediated by activities of genes grouped in clusters (*ipfABHI, ipfF, ipfDE*) and separated by IS*6100*-like sequences [23]. It is initiated by a CoA ligation to IBU encoded by *ipfF*, followed by aromatic ring activation by a dioxygenase (*ipfAB, ipfH, ipfI)* to produce 4-isobutylcatechol and propionyl-CoA, which then undergo further degradation. In *R. wittichii*, the propionyl-CoA molecule generated at this point has been described to be metabolised by the *pcc* operon (*pccA-pccB*) encoded at the chromosome as part of the central metabolism of the bacterium [26]. The cathecol-2, 3-dyoxygenase (CDO) activity of *ipfL* then catalyses the extradiolic cleavage of the 4-isobutylcatechol allowing the genes of the lower pathway (*ipfMNOPQST)* to participate in the downstream metabolism of the aliphatic intermediate. Several authors have shown that the *ipf* genes are located in a megaplasmid [24, 26]. In the strain MPO218 of *R. wittichii*, the *ipf* and other genes are associated with transposable elements and are located in the megaplasmid pIBU218 (~195 kbp), which has been proved to be conjugative to related strains such as *Sphingomonas granuli* TFA [23, 26]. This IBU-degrading strain was isolated and characterised in our group and used as a reference strain, as its genome has been sequenced and the complete genetic pathway of IBU degradation has been described [23, 26].

Other total or partial IBU transformation processes have been described using biochemical or proteomic approaches in strains such as *Variovorax* Ibu-1 [27] *Nocardia* sp. NRRL 5646 [28], *Patulibacter* sp. I11 [29] *Citrobacter freundii* PYI-2 or *Citrobacter portucalensis* YPI-2 [30], or cometabolism in the presence of other aromatic molecules is also the case of *Bacillus thuringiensis* B1 [31]. However, these pathways have not been genetically characterised, and the genes involved in IBU assimilation are unknown.

The use of bacterial communities for the degradation of various pollutants has been described as a more effective method than the use of isolated bacterial strains. This is due to the increased tolerance to environmental changes, the complementary interaction and cooperation among the components of the community and the greater resistance to the toxic effects of the pollutants and their derivatives [32–35]. Bacterial consortia capable of biodegrading IBU have also been reported in recent years [36–38]. Although some aspects such as the biodegradation efficiency of IBU or the description of bacterial communities using this compound as a carbon source have been studied [37, 39–41], there is still limited information on the evolution of the bacterial composition of the consortia [42], or the flow and exchange of genes involved in the process.

Here we have obtained two bacterial consortia capable of using IBU as the sole carbon source as a result of microbial enrichments from two wastewater treatment plants (WWTPs). In subsequent enrichments from one of these consortiums under that selective constraint, we recovered two re-enriched bacterial consortia that have an increased efficiency in the use of IBU. We have analysed the community composition of these consortia, and found that they all contain the genes that are involved in the IBU degradation pathway described previously [22, 26]. Our analyses showed that these genes that are located in plasmids and flanked by ISs, are likely mobilised within the microbial community. We also found that these genes show different expression patterns among the different consortia. Our results reinforce the importance of HGT in microbial ecology and will facilitate the rational design of biodegradation systems for IBU-contaminated wastewater based on natural or synthetic bacterial consortia.

## Materials and methods

### Enrichments of consortia and growth conditions

Consortia MPO977 and MPO984 were obtained by successive enrichments from the aeration tank of two WWTP at distant sites in Spain: Úbeda (Jaén) and Copero (Seville), respectively. Cultures in salt minimal medium (MM) [43] with IBU 1 g/L as carbon source were diluted one-fourth of the previous 20 ml saturated culture in aeration at 30°C for ~15 days, or until turbidity was clearly appreciated compared to a flask with no carbon source. The process was repeated for up to six months until differential growth was observed compared with the control flask. After culturing MPO977 in IBU plates, new enrichments were performed to give rise to 34ibu and 38ibu consortia. All consortia at the different stages were cryopreserved in glycerol (15%) at −80°C.

The generation time for growth was calculated as the slope of the line corresponding to the exponential phase of microbial growth (A600) on semi-logarithmic scale. To standardise the growth of the consortia, a small amount of the cryopreserved vial was inoculated as a 5 ml pre-inoculum in MM with IBU and incubated at 30°C at 180 rpm until late exponential phase (A600 = 0.8–1), which was reinoculated in a fresh medium at an A600 of 0.15 in a volume of 10 ml. Commercial IBU sodium salt was purchased from Sigma-Aldrich.

To determine the stability of the degradative phenotype of the stablished 34ibu and 38ibu consortia, serial sub-cultures were made in a rich non-selective medium (MML [43] diluted 50:50 in water) and the proportion of the population that retained the ability to use IBU as a carbon source was quantified at each sub-culture. For that purpose, independent cultures of each consortium were incubated in duplicate in 10 ml cultures of MM + IBU at 30°C until mid-exponential growth. After adjusting the OD to 1, 20 μl droplets of serial dilutions were plated on MM + IBU, diluted MML and MM without carbon source (control) and sub-cultured in diluted MML. For each subsequent passage in MML rich medium, cultures were successively diluted from saturation (OD = 1 approximately) to an OD of 0.15 in and re-diluted in the same medium until there was no growth on the MM + IBU plates, indicating loss of the ability to metabolise IBU.

### IBU quantification and metabolomic analyses

IBU concentration was determined with a Prominence UFLC XR SHIMADZU (Kyoto, Japan) HPLC. Chromatographic separation was performed at 30°C with a mobile phase of acetonitrile and 1% acetic acid (50:50 v/v) at a flow rate of 1 ml /min, using a Shimadzu VP-ODS C18 (4.6 mm × 15 cm, 5 μm) column. Detection was carried out by UV absorbance at 240 nm. For quantification, a calibration curve was built by injecting different volumes (7 points) of commercial IBU sodium salt in water at concentrations of 1, 0.1 or 0.01 mg/ ml. The retention time of IBU in these conditions was 12.718 ± 0.636 min. Linear regression statistics (consortia growth vs IBU concentration) were calculated using the “lm” function in ggplot2 [44].

Identification of intermediary metabolites in the IBU catabolism was performed using a non-targeted metabolomics approach using an Orbitrap Focus mass spectrometer (Thermo) coupled to an UHPLC-VaNquish system. Chromatographic separation was performed on a C18 column (2.1 mm × 100 mm, 1.6 μm) with solvent A (water, 0.1% formic acid) and solvent B (acetonitrile, 0.1% formic acid) at a flow rate of 0.4 ml /min. The gradient was programmed as follows: 5% solvent B at 0 min, increasing to 80% at 10 min, 100% at 15 min, held at 100% until 29 min, and returning to 5% at 30 min.

### DNA extraction and metagenome library construction

Total DNA from the consortia was extracted when reached OD of 0.7–0.8 growing on MM with IBU using the Wizard Genomic DNA Purification Kit, from Promega (Spain). Whole-metagenome shotgun was performed at Cabimer (Seville, Spain), following the Illumina Nextera DNA Flex Protocol, starting from 50 ng of total DNA. The pool of libraries to be sequenced was denatured and diluted to the concentration required to obtain 5 million filtered reads per sample. Cluster generation (monoclonal amplification) was performed on the Flow Cell NextSeq 500 MID-Output and 2 × 150 bp sequence length. A control library (PhiX) was always introduced in low proportion for quality monitoring.

We also used long-reads based metagenomic sequencing. High-molecular-weight DNA (2 g) was fragmented using a G-TUBE, followed by DNA damage repair and A-tailing using the NEBNext FFPE DNA Repair Mix and the NEBNext Ultra II End Repair/dA-Tailing Module. Barcode sequences were added, and sequencing adapters were ligated using the Non-Amplification Barcode Extension Kits 1–12 or 13–24. The library was then sequenced with a PromethION48 sequencer (Oxford Nanopore Technologies Limited).

Total DNA was also used for 16S rRNA gene amplicon sequencing. Libraries were prepared using the Nextera XT Index Kit for the addition of indices and adapters, followed by normalisation and pooling. The V3-V4 region of the 16S rRNA gene was amplified using the primers 5’-CCTACGGGNGGCWGCAG-3′ (forward) and 5’-GACTACHVGGGTATCTAATCC-3′ (reverse). Sequencing was performed on a NextSeq500 System (Illumina) (150 PE).

### Shotgun metagenome assembly and genome binning

The short raw reads were processed and cleaned as described previously [45]. Briefly, we removed the adapter sequences with Trimmomatic (v0.39) [46]. Those reads with Phred score < 30 and/or shorter that 50 bp were discarded using FastX-Toolkit (http://hannonlab.cshl.edu/fastx_toolkit/index.html). Raw Nanopore sequencing reads were processed by first removing adapter sequences using Porechop (v0.2.4c) (https://github.com/rrwick/Porechop), followed by quality filtering with Chopper (v0.8.0) [47], retaining reads with a minimum quality score of 9 and length above 500 bp. Filtered reads were then assembled using Flye (v2.9.3-b1797) [48] with metagenomic settings to generate preliminary assemblies. The Flye output also contains information related with the circularity of the replicons. Long-read alignments were conducted with Minimap2 (v2.28-r1209) [49] to map the quality-filtered reads to the assembly. These alignments were used as input for Racon (v1.5.0) [50]) to improve the assembly accuracy by iteratively polishing the consensus sequence. Bowtie2 [51] was employed to align short paired-end reads to the Racon-polished assemblies for a final polishing step with Pilon (v1.24) [52], providing additional refinement to the consensus sequences based on short-read alignments.

The contigs were subjected to multiple binning processes with MaxBin2 (v2.2.7, 46], MetaBAT2 (v2.15) [53], and Concoct (v1.1.0) [54]. Then, the recovered bins from each tool were used as input for de-replication and binning improvement with DAS-Tool (v1.1.2) [55]. To estimate the abundance of either bins, contigs or loci we used Bowtie2 [51]. We normalised the count of reads aligned to the reference contig by contig size, read length (150 nt) and sequencing depth (total number of reads per sample).

The assembled replicons were classified as chromosomal or plasmidic sequences with Plasmidhunter, which detect plasmids based on machine learning methods dependent on gene content profiles [56].

### 16S rRNA gene amplicon sequencing analyses

We generated an ASV table from the 16S rRNA amplicon data using the DADA2 [57] plugin from QIIME2 (v2023.8) [58]. We opted to only retain the forward reads to avoid biases due to the insufficient proportion of paired reads merged in the DADA2 step. The representative sequences were taxonomically annotated against the Ribosomal Database Project (RDP) (training set n° 19, 2023 release) [59] with the scikit-learn plugin as we previously did [60]. We create a ClustalW [61] alignment and Maximum Likelihood tree (1000 bootstraps) with MEGA X [62] to visualise the phylogenetic distance of the amplicon sequence variants (ASVs) to 16S rRNA gene sequences from type strains. The tree was re-rendered in iTOL (v7) [63].

### Quality control of bins and metagenome-assembled genomes, and taxonomic assignment

The binned genomes were surveyed with CheckM (v1.1.3) [64] and BUSCO (v5.4.3) [65] to measure contamination and completeness, respectively. We discarded bins >10% contamination and/or < 60% completeness. We used prodigal (v2.6.3) for gene-calling [66]. We created a consensus phylogenetic tree of the bins based on phylogenies of 92 marker genes with UBCG (v3.0) [67]. We investigated the taxonomy of each genome with GTDB-Tk (v2.4.0) (“classify_wf” command) [68]. This workflow uses the closest average nucleotide identity (ANI) value to place each genome into the closest species in the GTDB (r220). We curated the taxonomic assignment of metagenome-assembled genomes (MAGs) with the Type (Strain) Genome Server (TYGS) [69] and further investigation of valid names in the List of Prokaryotic Names with Standing in Nomenclature [69].

We investigated the taxonomic composition of the microbial communities with mOTUs3 [70]. This tool quantifies microbial community composition from metagenomic sequences by leveraging universal, protein-coding, single-copy phylogenetic marker gene sequences. We first splitted the Nanopore reads into 300 nt sized reads with “motus prep_long”, and we then used “motus profile” to characterise the communities (settings: ≥75 nt alignment, ≥3 marker genes cutoff).

### Nucleotide and amino acid sequence comparisons

*ipf* genes, and Ipf proteins in the metagenomic contigs and proteomes, respectively, were searched with local blastn and blastp tools using the *R. wittichii* megaplasmid pMPO218 sequences as a query [71]. We used “diamond” to search for Ipf protein homologs in external HQ-MAGs (e-value <0.01) [72]. These HQ-MAGs were obtained from the “Genomes from Earth’s Microbiomes (GEM) catalog” [73], which comprises 9143 genomes binned from diverse Earth environments, and 1083 genomes binned from long-read metagenomic sequencing data obtained from 23 WWTPs (activated-sludge) [74]. We also searched Ipf proteins within 699 973 plasmids, belonging to 214 950 plasmid taxonomic units (PTUs), available in the JGI-IMG/PR database [75].

To analyse the synteny of the plasmids used in this work, we first annotated the DNA sequences with prokka [76], using a dataset of trusted *ipf* proteins and related orthologs found in the sequences (File S1) (min evalue =1e-20). We then used GenoFig (v1.1) [77] to visualise and label the syntenic genes.

### RT-qPCR assays

RNA extraction from mid-exponential phase consortia cultures grown in MM with either IBU 1 g L^−1^ or ACE 2 g L^−1^ was performed out as previously described [78]. After DNase I treatment with a DNA-free kit (Ambion), the RNA was purified using RNAeasy columns (Qiagen). The absence of contaminating DNA was confirmed by PCR amplification of *ipfA* gene with primers ipfA-Fw and ipfA-Rv. Reverse transcription (RT) of total RNA (2 μg) was carried out using the High-Capacity cDNA reverse transcription kit (Thermo Fisher Scientific), followed by clean up using the QIAquick PCR purification kit (Qiagen), according to the manufacturer’s indications. Target cDNAs (10 ng) were amplified in triplicate in separate PCR using 0.3 μM of each primer (Table S5), and a standard curve was made using serial dilutions from 25 ng to 0.0025 ng of *R. wittichii* MPO218 genomic DNA to estimate the relative abundance of transcripts. A melting curve analysis was performed to identify non-specific amplification. The results are the average of three technical and at least 2 biological replicates. Significance of the differences reported was assessed using the Student's t-test for unpaired samples not assuming equal variance.

## Results

### Obtaining enriched consortia able to use IBU as the sole carbon source

**Figure 1:**
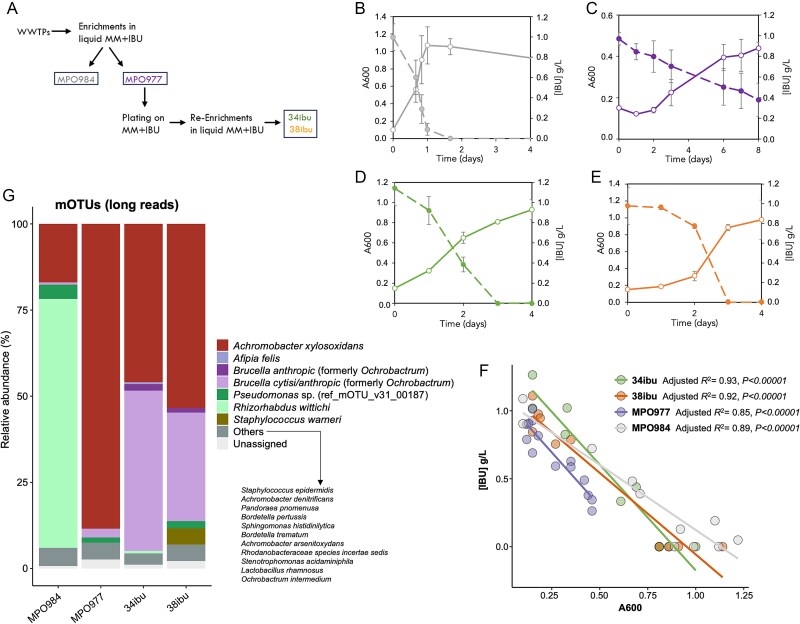
**Characterisation of IBU-degrading consortia MPO984, MPO977, 34ibu and 38ibu**. **Panel A**) schematic representation of the enrichment process for the generation of the consortia. MPO977 and MPO984 come from WWTPs in Seville and Úbeda, respectively. **Panels B-E**) IBU biodegradation and growth rate of the consortia. Growth curves of the consortia MPO984 (B), MPO977 (C), 34ibu (D), and 38ibu (E) in MM with IBU as sole carbon and energy source (white circles and solid lines). Depletion of IBU over the time in the cultures (dashed lines and solid circles). The graphs show the mean and standard error of three independent cultures. **Panel F**) correlation of IBU depletion associated with growth of each consortium. Each dot represents a single measurement of both IBU and biomass absorbance. **Panel G**) taxonomic composition of the microbial consortia. The abundance of bacterial species within each microbial consortium was determined by analysing the reads mapped to marker genes in the mOTUs database. The remaining composition that could not be mapped is categorized as “others”.

In this work we have selected several IBU-degrading consortia at different stages of an enrichment process. The initial cultures, named MPO977 and MPO984, were obtained through enrichment cultures in a defined medium containing IBU as the sole carbon source for several weeks using samples taken from the effluent of the secondary digesters of two WWTPs in two cities in Spain (Úbeda and Seville, respectively). When turbidity was detected in the presence of IBU, they were re-diluted in the same medium for ~2–3 months. In a second stage, they were plated onto solid medium and incubated for 2–3 weeks, to isolate bacterial colonies that could independently metabolise the drug completely. However, the isolation of single colonies able to use IBU as the sole carbon source was not possible as communities that collectively participated in biodegradation were always detected (Fig. S1). To improve IBU assimilation, two of these communities (34ibu and 38ibu) obtained from MPO977 were selected, re-enriched in liquid cultures 3–4 additional times and used for characterisation in subsequent experiments (Fig. 1A). The composition of the four communities has been studied and their IBU-degrading capacity has been analysed. Doubling times for each consortium were 6.75 h^−1^ for MPO984, 80.78 h^−1^ for MPO977, 36.43 h^−1^ for 34ibu and 28.47 h^−1^ for 38ibu (Fig. 1B-D). The growth rate of the MPO984 consortium with IBU as the sole carbon source was the highest, reaching stationary phase within 1 day of incubation (Fig. 1B). In contrast, consortium MPO977 showed a much lower growth rate, reaching stationary phase after 8 days (Fig. 1C). This consortium reached an optical density of only 0.45 as it was unable to uptake all the IBU present in the medium. Subsequent enrichments of this consortium, after plating on a solid medium containing IBU, resulted independently in the two degrading consortia 34ibu and 38ibu. These re-enriched consortia exhibited an improved growth rate (Fig. 1D and E), indicating an optimisation of (or adaptation to) the IBU assimilation process. Additionally, this result suggests differences in composition and/or genetic information organisation of the improved consortia 34ibu and 38ibu compared to their parental consortium.

Quantification of the IBU concentration in the culture medium for each of the consortia showed that it was inversely proportional to the increase in culture turbidity (Slope = −0.96; adjusted *R*^2^ > 0.85; *P*-value <0.00001) (Fig. 1F), indicating that IBU was used as the source of carbon and energy to drive the increase in bacterial biomass. Although the final biomass yield for both consortia was similar, reaching an optical density of 1, the growth rate and IBU removal rate of the 34ibu consortium was higher than that of the 38ibu, so they were treated hereafter as independent degrading consortia. The strain MPO218 loses the ability to use IBU when grown in the absence of selective pressure [23], mainly due to recombination between the IS*6100* copies flanking *ipf* genes. To test whether this also occurs in enriched consortia, we performed successive cultures in non-selective rich medium and we analysed, in each subculture, the ability to use IBU. The results demonstrate that this capacity is lost after 20–70 generations, which shows that either species or genes essential to this process disappear from communities when selective pressure is released.

### Insights into the IBU-degrading communities

The metagenomes of the IBU-degrading consortia were analysed to identify the microbial populations that can use IBU as a carbon and energy source. The consortia showed low species diversity overall (Fig. 1G). *Achromobacter xylosoxidans* emerged as the dominant species in the consortium MPO977 and it also appeared in MPO984 with a relative abundance of 17%, although this sample came from an WWTP from another geographically distant city. In MPO984, *R. wittichii* was identified as the dominant species. Successive enrichments of MPO977 to generate 34ibu and 38ibu resulted in a shift in the composition of these consortia. *A. xylosoxidans*, initially the most abundant species in MPO977, significantly decreased in relative abundance, whereas *Brucella anthropi* (formerly *Ochrobactrum anthropi*), initially present in minor quantities, became more prevalent in the improved communities (Fig. 1G). These compositional shifts in the consortia possibly led to an increased ability to assimilate IBU, with an improved metabolic rate (Fig. 1F).

To further elucidate community dynamics, we performed 16S rRNA gene amplicon sequencing to monitor strain-level changes during the enrichment process. These analyses also revealed low bacterial diversity, with 304 ASVs in the dataset, of which only nine had an abundance greater than 1% in any of the communities (Fig. S2, Table S1). Among the identified ASVs, *Achromobacter* was represented by four distinct ASVs, two of which were consistently rare across all samples. Similarly, 19 ASVs were classified within the order *Sphingomonadales*, with only five of them being highly abundant, and 24 ASVs were classified as *Sphingomonas* (likely an undescribed genus related to *Sphingomonas* and *Rhizorhabdus*), of which only five showed high abundance in the MPO977 and MPO984 samples (Fig. S2, Table S1). These patterns further support the presence of genotypic variability (strain diversity) within closely related taxa, likely reflecting adaptations to the selective pressures imposed by the enrichment process. In contrast, *Brucella* was represented by a single ASV, which increased significantly during enrichment, indicating low strain diversity within this genus. *Caenibius tardaugens* was also identified in the enriched consortia, but it also exhibited limited strain diversity (Fig. S2). Finally, a group of ASVs within the family *Erythrobacteraceae* increased by ~5-fold in the 34ibu and 38ibu samples. None of the above 24 ASVs classified as *Sphingomonas* were detected in the enriched consortia (34ibu and 38ibu) (Fig. S2, Table S1).

### Long-read based metagenomics enables the recovery of plasmids with IBU catabolic genes

An important aim of this work was to identify the members within the microbial consortia and to locate those genes that might be involved in the IBU assimilation. We were also interested in the evolutionary movement of these genes associated with the presence of the drug as selective pressure. To this end, the metagenomes of the consortia were processed to reconstruct the genomes of their components and to study their IBU catabolic genes. The long-read assembled metagenomes for each sample were small in size (46.5–74.4 Mbp per sample) (Table S2), which is congruent with the low diversity bacterial community found in the samples (Fig. 1G). The read coverage of the assembled was high (97–250 coverage mean per sample), keeping a low assembled fragment count (616–2296 assembled fragments per sample) (Table S2). The binning process yielded 37 bins of different qualities (Table S3). To further investigate the presence of IBU catabolic enzymes, we opted to retain redundant bins to avoid forfeiting opportunities to discover mobile genes. We were successful in the MAGs recovery for the most abundant species in our consortia (*A. xylosoxidans*, *R. wittichii,* and *B. anthropi*) as evaluated through the profile of marker genes (mOTUs) (Fig. 1G).

**Figure 2:**
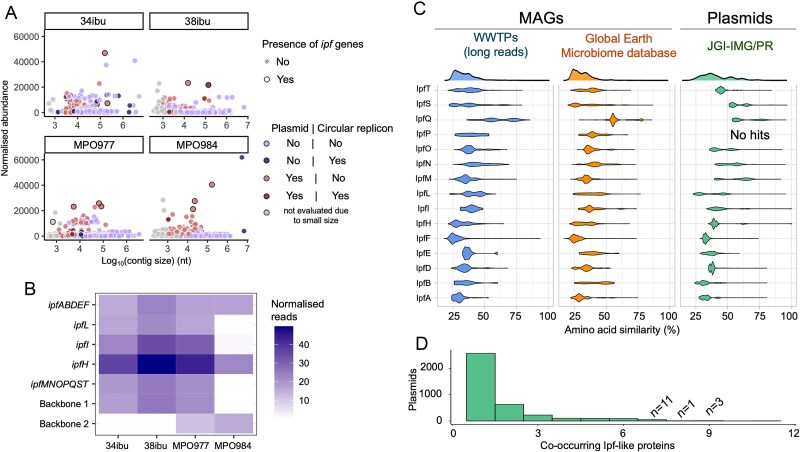
**Distribution and representation of *ipf* genes in metagenomes. Panel A**) scatter plot of the contigs assembled from the metagenomes. Each dot represents a contig and shows its normalised read abundance (reads per million, resulting from contig-length normalization followed by sequencing depth normalization and relativized to 1 Mbp) in each sample (Y axis) and its length (Log_10_[contig size]). Replicons containing *ipf* genes are marked with an outer circle*.* The colour code represents both the circularity of the replicon and its type—plasmid or chromosome—according to the colour legend. **Panel B**) relative abundance of the *ipf* genes described for *R. Wittichii* pMPO218 plasmid among the different consortia. Heatmap representation of the relative abundance of the *ipf* genes flanked by IS*6100* of the upper (*ipfABDEF, ipfI, ipfH* and *ipfL*), lower (and *ipfMNOPQST*), and representation of backbones type 1 and 2 in the metagenomes of consortia 34ibu, 38ibu, MPO977, and MPO984. The relative abundance of these backbones was estimated based on a conserved region of each plasmid that did not contained transposases nor *ipf* genes. The relative abundance (%) was normalised by each region size and sample sequencing depth. **Panel C**) distribution of sequence similarity search hits of Ipf proteins in metagenome-assembled genomes (MAGs) recovered from metagenomes obtained from wastewater treatment plants (1083 MAGs) (left side) [74], 9143 MAGs from diverse environmental metagenomes (Centre) [73], and in a database of 699 973 plasmids, belonging to 214 950 plasmid taxonomic units (PTUs) (right side) (IMG/PR database; [75]). **Panel D**) count of co-occurring proteins sharing significant similarity to Ipf proteins in the IMG/PR database. The observed low frequency of co-occurring Ipf proteins in within plasmids suggests that the entire IBU biodegradation machinery is not commonly mobilised within a single plasmid, which might imply potential requirement for a synergistic microbial community to effectively metabolise this drug.

The metagenome contigs and bins were analysed to locate all the *ipf* genes which were previously described as responsible of IBU degradation [22, 25–27]. We detected diverse copies of these genes, sharing nearly 100% similarity with the described *ipf* genes. However, we found these genes exclusively in plasmid sequences, including some large circular assembled replicons, rather than in binned chromosomal contigs (Fig. 2A). Plasmids are the main drivers of HGT between bacterial cells, and our results suggest that *ipf* genes could be involved in these events within the microbial consortia capable of thriving in a culture medium with IBU as the sole carbon source. Also, the relative abundance of these genes varied considerably within samples (Fig. 2B), implying that they are not consistently located in the same host cell. This observation may also explain the requirement for cooperative microbial consortia to complete the entire IBU catabolic pathway and the impossibility to isolate single strains capable to growth solely in media containing IBU as carbon source (Fig. S1). Given the large size of the plasmids found, it seems unlikely that these are high-copy number plasmids that would explain the differences in relative abundance [79].

Based on our results, we aimed to determine the distribution and co-occurrence of IBU catabolic genes by microbial replicons in nature. To this end, we searched for the proteins encoded by these genes within a database of 9143 high-quality MAGs (HQ-MAGs) binned from diverse environmental metagenomes [73], another database of 1083 HQ-MAGs assembled from long-read sequences from 23 WWTPs [74], and a database of 699 973 plasmids, belonging to 214 950 plasmid taxonomic units (PTUs) [75]. Overall, a low number of hits shared a high identity to Ipf proteins (Fig. 2C). The Ipf proteins also showed a dispersed distribution in terms of sequence similarity to the target proteins, suggesting that the *ipf* genes could have not co-evolved in the same replicon. Indeed, although those hits to the plasmid database show a slightly higher similarity than those of the MAGs, most of them just have homologs for 1 out of the 15 Ipf proteins, and any of them had hits for the >11 Ipf proteins (Fig. 2D). Our results indicate that these proteins are not highly conserved nor commonly co-selected across Earth microbiomes.

### Dispersion and evolution of the *ipf* genes in IBU-degrading consortia

**Figure 3:**
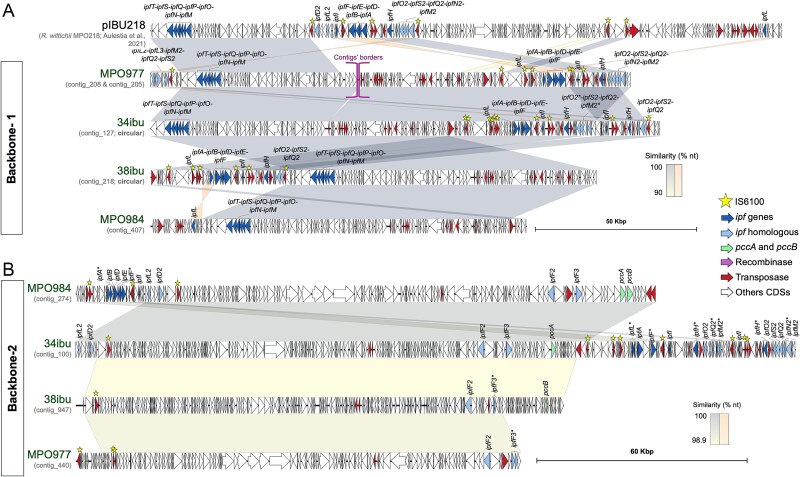
**Genetic organization of syntenic and evolved plasmids with *ipf* genes.** This figure highlights the structural organization of plasmids grouped into two distinct backbones: “Backbone-1” (**Panel A**) and “Backbone-2” (**Panel B**). These plasmids exhibit extensive synteny, with large DNA regions sharing high nucleotide similarity (visualised as a yellow-to-grey gradient, per the legend). The nucleotide similarity of inverted regions is indicated in orange gradient. Coding sequences (CDSs) are color-coded based on their functional categories, and all *ipf* genes and their homologous sequences are labelled with gene names. Stars denote transposases classified as “IS*6100* transposases”. To facilitate visualisation of synteny, contigs_205 and contig_208, which are non-circular, are displayed together. The plasmid pIBU218 is included as a reference within Backbone-1 to demonstrate mobilisation events involving *ipf* loci. Each plasmid or contig is labelled according to the sample from which it was assembled, irrespective of its relative abundance (see Fig. 2B). Additional mobilisation events involving *ipf* loci across other replicons are detailed in supplementary material (Fig. S3).

The analysis of the contigs obtained from the long-read sequencing of the consortia revealed the presence of the *ipf* genes involved in the IBU degradation in the *R. wittichii* megaplasmid pIBU218 [23], which shares a high nucleotide similarity with several plasmids recovered from our metagenomes. However, the sequences of the plasmids in the four consortia did not share the same backbone as pIBU218. Specifically, we found plasmids with two alternative backbones containing different *ipf* genes organisations with distinct relative abundance and distribution, referred to here as Backbone-1 (Fig. 3A) and Backbone-2 (Fig. 3B). However, as in pIBU218, both plasmid versions also contain several copies of the IS*6100* insertion sequence.

Backbone-1 plasmids are found in high relative abundance in MPO977 and its derivatives containing the same *ipf* genes of the upper and lower IBU degradation pathways as pIBU218 (Fig. 2B and 3A) [23, 26]. In the process of evolution of MPO977 to obtain 34ibu and 38ibu, there have been several major changes affecting the plasmid backbones and the distribution of the *ipf* genes. In this new conformation, the upper and lower pathways have undergone various rearrangements, and in 38ibu are in inverted orientation and are now closer together. Also, in the case consortia 34ibu, we found two copies of *ipfI* and *ipfH*, that are not present in the parental consortium MPO977, nor the other evolved 38ibu. Backbone-1 is also present in the consortium MPO984, but at a very low level of representation and only carries genes of the lower pathway (Fig. 2B and 3A). Contig_127 from 34ibu consortium, and contig_128 from 38ibu, are closed plasmids carrying all *ipf* genes of the upper and lower pathways, and have been named plasmids pIBU219 and pIBU220, respectively in this work.

The most abundant plasmid found in MPO984 was of the type Backbone-2, which carries genes involved in the upper pathway (*ipfABDFE, ipfI*) (Fig. 2B and 3B). No genes for the lower pathway (*ipfMNOPQST*) were detected in this prevalent plasmid within the consortium. This backbone is also present in MPO977 but nearly absent in its derivatives, 34ibu and 38ibu (0.01% of the metagenomic reads) (Fig. 2B), indicating its abundance has been decreased during the enrichment process of the former. This low represented plasmid of the Backbone-2 type was found in the MPO977 and 38ibu consortia, and contained no *ipf* genes but just orthologs with low amino acid similarity (labelled as *ipf2/3* in Fig. 3B). We cannot rule out that, given that these unabundant contigs are unclosed assemblies of plasmids, other *ipf* genes could also be in the surrounding locations of the replicons. As this region was in synteny with that of MPO984, we interpreted that the polished assembly might not be of enough quality for the gene calling. Therefore, we analysed in detail their nucleotide sequences, and found it aligns correctly with all the *ipfABDFE* genes (data not shown). We also observed the lack of SNPs in the *ipfABDFE* cluster in 34ibu. For MPO984, only two *ipf* genes contained SNPs, ipfA^*^ (3 missense variants) and in ipfF^*^ (1 missense variant) (Table S4), although we have no evidence that these mutations lead to loss of function of these genes.

An interesting gene mobilisation event was the detection of the *pccAB* genes in plasmids of type Backbone-2 (Fig. 3B). *pccAB* encodes the propionyl-CoA carboxylase, which is involved in the incorporation of propionyl-CoA into the branched-chain amino acids, odd fatty acids, and branched-chain fatty acid catabolic pathways found in a wide variety of organisms (including humans) [80], and has been found to be necessary for complete growth in IBU [26]. These activities are mostly found encoded in the chromosome of the host and rarely found in plasmids (NCBI database). The need for co-evolution of both sets of genes for IBU degradation could be confirmed by the identification of these genes in plasmids together with the *ipf* genes.

Other plasmids with different structures, containing only a few *ipf* genes, were assembled in addition to the Backbones −1 and − 2 (Fig. S3). We have also identified several less-conserved orthologous genes to the *ipf* genes within most of the analysed plasmids (here named as *ipf*-2/3) (Fig. 3A and B, Fig. S3, File S1), although we cannot attribute their involvement in IBU catabolism with current evidence.

Our results demonstrate the mobilisation of *ipf* genes within and between plasmids recovered from different locations, but to provide more evidence to this phenomenon, we searched for other replicons that contain *ipf* genes in IBU degrading organisms obtained from other locations. This is the case of pIBU218, obtained from a *R. wittichii* strain isolated from a WWTP in Barcelona (Spain) [23] and pRW412a, isolated from *S. granuli* RW412 at the Elbe River (Germany) [24] (Fig. S3). Beyond, we found closely related replicons that have not acquired *ipf* genes or might have lost them, such as the plasmid p1 from *Sphingobium* sp. CAP-1 [81] (Fig. S3). Overall, our results demonstrate that *ipf* genes are likely being mobilised among different replicons and species, keeping their nucleotide sequence almost invariable.

### Regulation of *ipf* genes expression has evolved throughout the consortia

To gain insight into the IBU biodegradation and assimilation rates during consortia evolution, we investigated the expression pattern of specific genes that play an important role in the upper and lower pathways in response to the drug. As reference strain, we used *R. wittichii* MPO218, whose gene organisation and sequence had been described [26], although the regulation of their expression was poorly understood. For this study, consortia MPO984, MPO977, 34ibu, and 38ibu were grown to mid-exponential phase in a non-inducing medium with acetate (ACE) as a carbon source and a medium containing IBU as carbon source under the same conditions. As genes involved in the degradation of the upper part of the pathway, we studied the expression of the genes *ipfF*, *ipfA,* and *ipfI,* which are involved in the upper pathway for the assimilation of IBU (Fig. 5A) and encoding the activities Ibuprofen CoA ligase (ICL), Ibuprofenyl-CoA 1,2 dioxygenase subunit Alpha (ICD), (2Fe-2S)-binding protein, respectively. For the lower pathway, the genes *ipfL*, *ipfM,* and *ipfQ* encoding the CDO, 5-carboxymethyl-2-hydroxymuconic-semialdehyde dehydrogenase (HMSD) and 2-oxo-3-hexenedioate decarboxylase activities, respectively were selected (see Fig. 5A). The *ipf* genes of the upper pathway were selected because they are organised in distinct gene clusters in the reference plasmid pIBU218 and flanked by IS*6100* sequences. They all encode critical catalytic activities in the IBU degradation pathway.

**Figure 4:**
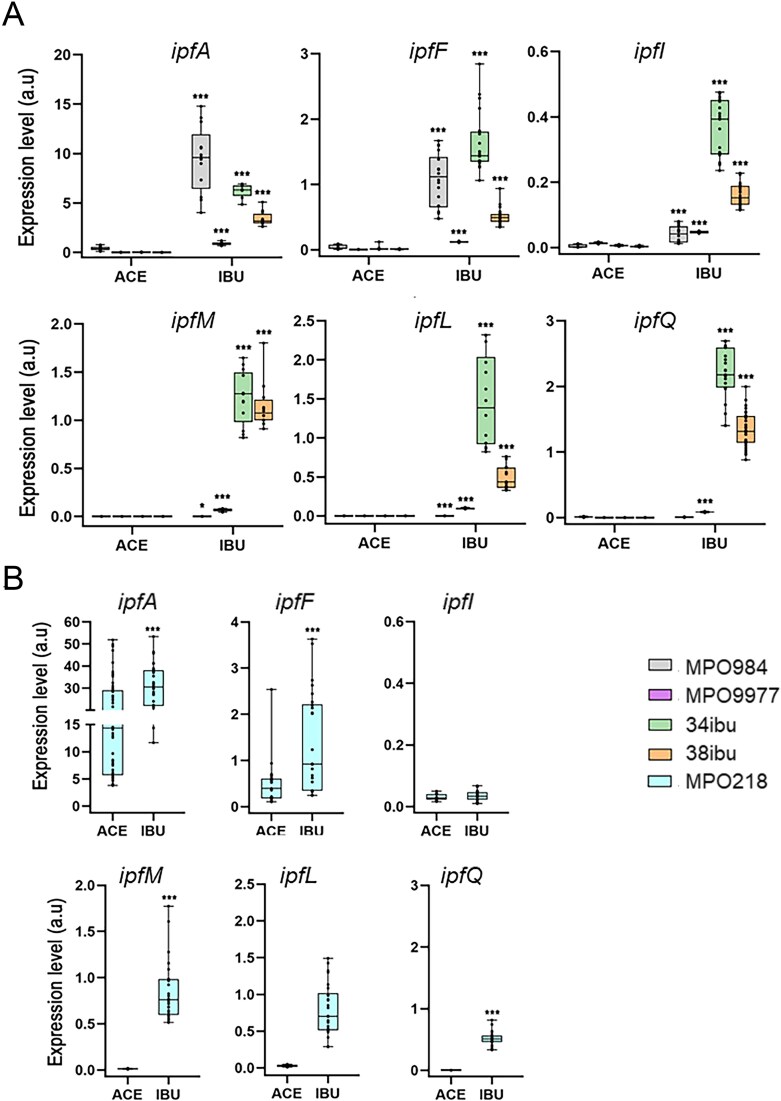
**Expression levels of selected *ipf* genes from the upper and lower pathway. Panel A**) quantification by RT-qPCR of the expression levels of *ipfA*, *ipfF* and *ipfI*, from the upper pathway, and *ipfM*, *ipfL* and *ipfQ*, from the lower, for cultures of consortia MPO984, MPO977, 34ibu and 38ibu growing in MM with IBU or ACE as sole carbon and energy source. **Panel B**) expression levels of the *ipf* genes for strain MPO218 (blue) growing in IBU or ACE. Data are expressed in arbitrary units (a.u.). Stars designate *P* values for the Student's t-test for unpaired samples not assuming equal variance (^*^P < .05; ^**^P < .01; ^***^P < .001).

Our results show a significant induction (although to a different extent), in the presence of IBU compared to the non-inducing medium (ACE) for the upper pathway in the four consortia (Fig. 4A, top panel). Focusing on the parental consortia MPO984 and MPO977, we detected the induction of the *ipf* genes (shown as expression ratio in IBU vs. ACE) for the upper pathway *ipfA*, *ipfF,* and *ipfI* (23-, 26- and 8-fold, in MPO984, and 57-, 28- and 4-fold in MPO977, respectively). However, upon enrichment of the MPO977 consortium to the 34ibu and 38ibu consortia, the increase in the ability to induce the expression of genes involved in the upper pathway is magnified up to levels of 280-, 98-, and 61-fold for 34ibu, and 360-, 48- and 51-fold for 38ibu, respectively for *ipfA, ipfF,* and *ipfI*. The induction of the upper pathway in the MPO218 strain carrying the pIBU218 plasmid, which does not have the same backbone as the consortia (see above) is very low or zero (1.6-, 2.9-, and 1.2-fold induction for *ipfA*, *ipfF,* and *ipfI*) (Fig. 4B). Nevertheless, this effect is partly because in strain MPO218, in contrast to the consortia, *ipfA* seem to be constitutively active, as expression levels in ACE are as high as those in IBU.

The expression of the lower pathway for IBU biodegradation seems to follow the same pattern as the upper pathway in the consortia 34ibu and 38ibu compared to their parental MPO977. There is a clear induction of *ipfM*, *ipfL,* and *ipfQ* in the presence of IBU, which coincides with an improved ability to use IBU as carbon source (Fig. 1). Nevertheless, the expression levels of the genes involved in the lower pathway in MPO984 are almost undetectable near zero, both in induced and non-induced conditions, which is consistent with the very low representation of these genes among the sequences identified in the MPO984 consortium (Fig. 1G).

### Metabolite identification for the IBU assimilation

**Figure 5:**
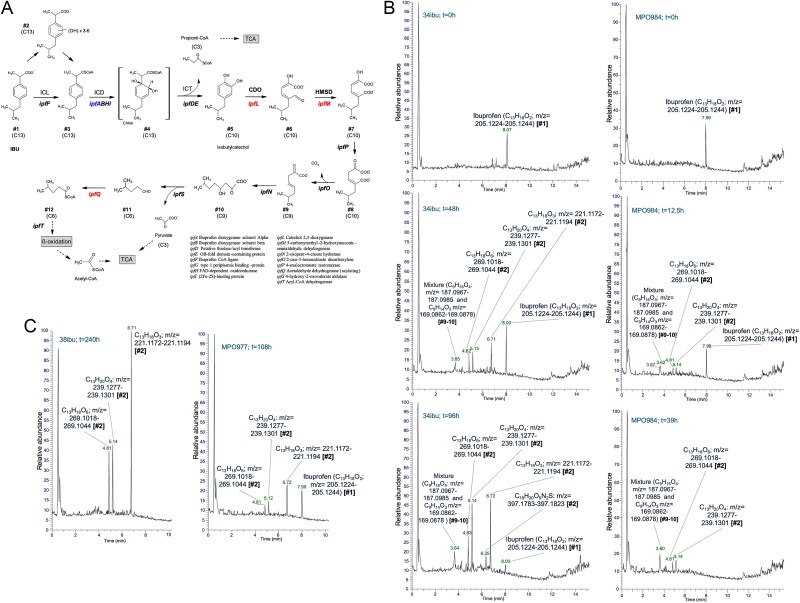
**Analysis of the metabolites from IBU assimilation in the consortia. Panel A**) proposed IBU biodegradation pathway for the IBU -degrading consortia. The genes whose expression was studied in this work for the upper pathway are *ipfA*, *ipfF* and *ipfI*, and the ones for the lower, *ipfM*, *ipfL* and *ipfQ*. The activities encoded in the *ipf* genes are shown in the lower part. **Panel B**) relative abundance of the metabolites identified for 34ibu and MPO984 at different time points. The major peaks at each time are labelled with the retention time along the X-axis, the molecular weight, and an assigned metabolite number (#) that corresponds to those in panel A). The Y-axis represents the relative abundance for each peak. Sample 34ibu and MPO984 are represented at three time points, according to the arrows in Fig. S4. **Panel C**) metabolites detected at the last time point for MPO977 and 38ibu. The metabolites at earlier times were coincident with those of 34ibu, and only the last point is shown for simplicity.

The metagenomic analysis together with expression analysis and growth rate of the consortia showed similar genomic content for MPO977 (upper and lower pathways), with rearrangements in its 34ibu and 38ibu derivatives, and adaptation in the induction of its gene expression, leading to an improved ability to use IBU. Conversely, consortium MPO984 contains only a high relative abundance of the upper pathway, but a very low relative abundance of the lower, if present at all, i.e. not detectably expressed. To investigate if the MPO984 used an alternative lower pathway we searched for the metabolites produced during the assimilation of IBU at three points of the growth curve. We obtained the metabolomic profiles from samples recovered at: (i) the starting point of the culture, (ii) mid-exponential growth, and (iii) end of the exponential growth (Fig. S4). We aimed to investigate the similarities and differences in the metabolic activities between the different consortia, and observed that the main metabolic profiles were highly similar in all communities (Fig. 5). Based on the masses found in our data, our results suggest a poly-hydroxylation of the IBU ring (metabolites C_13_H_18_O_3_, C_13_H_20_O_4_ and C_13_H_18_O_6_), with certain ability to further complement the ring activation by the addition of the -COSCoA residue in either non-hydroxylated or polyhydroxylated intermediates (Fig. 5). These forms remained present at stationary phase in the four consortia, even when IBU was totally consumed. The IBU molecule was only detected in consortium MPO977, which correlates with the partial assimilation detected (Fig. 1C). In MPO977 and its derivatives 34ibu and 38ibu, we also detect accumulation of a probable fragment of the -COSCoA containing (polyhydroxy) ibuprofen (i.e. C_19_H_30_O_5_N_2_S (m/z = 397.1783–397.1823)), showing that addition of -COSCoA could proceed using IBU or its hydroxylated derivatives as substrates. We also detect accumulation of metabolites in the lower pathway, after the meta-cleavage of the aromatic ring and decarboxylation of the intermediates, (i.e. mixture of C_9_H_14_O_3_, (m/z = 187.0967–187.0985) and C_9_H_14_O_3_ (m/z = 169.0862–169.0878)) (Fig. 5B), showing a kinetical misbalance in the reaction.

 Altogether, most metabolites identified during the growth curve of the consortia are coincident in consortia MPO977, 34ibu, 38ibu, and MPO984, although they differ in their relative abundance. We detect polyhydroxylated forms of IBU accumulated in the mid-exponential phase of the growth curve, to which a -COSCoA residue is subsequentially added by a CoA ligase (encoded by *ipfF* or other orthologs) to be cleaved of the aromatic ring at the *meta* position. The metabolites detected from the activities of the lower pathway are consistent with the metabolic pathway previously proposed [26] for all the consortia analysed.

## Discussion

We have explored the presence and the evolution of the species that comprise the different consortia in a medium containing IBU as sole carbon source. The most abundant species detected were *A. xylosoxidans*, present in all consortia but predominant in MPO977 and its derivatives, and *R. wittichii*, most abundant in MPO984. *B. anthropi* was found to be predominant in consortia 34ibu and 38ibu, whose ability to use IBU efficiently was improved after further enrichments of MPO977. Other genera, such as *Pseudomonas*, was detected in the four consortia at low abundance (Fig. 1G). All these species or genera identified in the consortia have previously been described to be involved in the biodegradation of environmental pollutants [82–91], but to our knowledge, only *R. wittichii*, and recently, one species of *Pseudomonas* have been reported to be able to grow using IBU as sole carbon source [23, 92]. There are, however, reports of microbial consortia growing on IBU [36, 37, 93], and a few report the presence of some of the less abundant taxa in our consortia (i.e.: *Pseudomonas* or *Achromobacter*) in IBU-mineralising consortia [36, 38, 42]. Additionally, it is interesting that the original consortium MPO977 was not able to completely degrade the IBU present in the medium, whereas its derivatives, 34ibu and 38ibu, were able to do so. This is probably due to a change in the species composition of the consortium and/or a redistribution of the genetic information required for IBU biodegradation during the enrichment process, selectively favouring taxa or replicons initially present in the WWTP samples at very low abundance, and potentially capable of degrading compounds structurally similar to IBU. In all cases, the metabolites detected for the assimilation of IBU comprise polyhydroxylated forms of IBU prior to the activation via the –COSCoA addition. There are precedents for the utilisation of trihydroxylated intermediates in the degradation of aromatic compounds via meta ring-cleavage by *Burkholderia* [94] and *Variovorax* [95], and specifically for IBU in a *Variovorax* strain [27], but had not been reported for the strains bearing the *ipf* genes.

Our attempts to isolate strains capable of independently growing on IBU were unsuccessful. Despite identifying plasmids carrying all the *ipf* genes—sufficient in theory to metabolise IBU into more accessible intermediates—other factors may explain this outcome. Although we detected key intermediary catabolites (Fig. 5), it remains possible that low-abundance toxic IBU derivatives exist, which might only be neutralised in the presence of other microorganisms. In any case, the upper and the lower catabolic pathway of IBU are not located in a single replicon in MPO984 (Fig. 3), and the lower pathway is almost absent in this consortium (Fig. 2B). This suggests that the observed interdependence among species within the consortia could be a key element in their ability to process IBU, where microbial cooperation enhances the overall catabolic efficiency. Such interactions, although rarely described in ecological contexts [96] align with the, Black Queen Hypothesis [97], in which species rely on each other for the completion of metabolic processes. The failure to isolate single strains suggests that the consortia function more effectively as a cooperative unit [98, 99], facilitating the degradation of IBU. Nevertheless, given the mobility of *ipf* genes, we hypothesise that these genes might eventually integrate into an optimal genomic context, enabling complete IBU utilisation as a carbon source [100] although this may require new enrichment and selection processes with further evolution of the consortia.

A detailed study of the location and abundance of the *ipf* degradation genes show differences during the enrichment of the consortia (Fig. 3). Our results indicate that the *ipf* genes, present in the original consortium in MPO977, have been rearranged during the selection process to generate consortia 34ibu and 38ibu. These genes, and the plasmids bearing them, were found in varying abundance within the obtained consortia (Fig. 2B). Its interpretation shows that several genetic rearrangements appear to have taken place, as evidenced by differences in nucleotide sequences between the gene clusters of the upper and lower pathway, and the duplication of certain *ipf* genes (Fig. 3). We cannot rule out that contigs_205 and contig_208 of consortium MPO977 were in one larger molecule (we were not able to close the plasmid in this consortium), and during the evolution to 34ibu and 38ibu, the upper and lower genes have rearranged to yield closed plasmids that assimilate IBU more efficiently than the parental one. This rearrangement may also lead to a more efficient way of expressing these genes by optimising the induction in the presence of IBU. The induction rate of the upper pathway in MPO977 (57- fold) seem to be sufficient to allow its growth on IBU as carbon source. In *R. wittichi* the induction rate of the upper pathway is also low (1.6-fold) although int this case the expression levels of *ipfA*, *ipfF,* and *ipfI* seem to be constitutively expressed in non-induction conditions (Fig. 4B). In contrast, consortium MPO984, presents a great induction of the upper pathway, but low relative abundance or no expression of the lower IBU degradation pathway. This constitutes one of the most surprising results, because this consortium presents the highest growth rate of all using IBU as carbon source. This community does have and effectively express the upper pathway (*ipfF-ABHI-DE)* genes involved in the IBU catabolism into 4-isobutylcatechol, and the metabolites identified show similar intermediates as in the consortia MPO977, 34ibu and 38ibu. This suggests that consortium MPO984 contain similar activities but encoded by other genes different to *ipfMNOPQST*, leading to the efficient assimilation of IBU.

The most abundant *ipf* genes identified in the MPO977, 34ibu and 38ibu consortia are part of a plasmid type we have named Backbone-1. Similarly, the upper pathway genes in the MPO984 consortium are part of a second plasmid type, which we have termed Backbone-2. However, the *ipf* genes found in pIBU218 [26], and those contained in plasmid pRW24a between the upper and lower pathways [24] isolated from *R. wittichii* and *S. granuli,* respectively, are located in a different backbone to the ones described here. This suggests that the IBU degradation genes, in species from different origin, are somehow recruited on different plasmid backbones, and evolved and selected in nature under restrictive conditions. This demonstrates the mobilisation of IBU catabolic genes among different replicons and species. In this line, we have also found evidence of *ipf* mobilisation between different replicons (Fig. S3). A mechanism of homologous recombination between different copies of IS*6100* together with chromosomal rearrangements occurring during transposition has been proposed to play an essential role in the recruitment of *ipf* genes in all these plasmids [26]. Furthermore, the relative positions of the *ipf* genes and IS*6100* copies are similar in all plasmids, indicating that this arrangement of sequences was already present in the consortia in the environment and are not a consequence of the selection process in the laboratory. Nevertheless, these processes of rearrangement and gene mobilisation must also have contributed to the selection of the consortia, and the evolution of these, to generate more efficient degrader communities. For the MPO984 consortium, we have identified plasmids that only carry the *ipf* genes of the upper pathway, so it is likely that genes other than *ipf* are involved in the lower pathway. These may be present in the same strain carrying the plasmid or in a different one. We cannot rule out the possibility that, as in MPO984, the other consortia may also contain genes other than *ipf* that may be involved in the IBU catabolism. These may contribute to the degradation process to a different extent. In fact, some genes that have low similarity to the *ipf* in IBU-degrading consortia has been described in the literature [92]. Other *ipf*-like genes, whose function has not yet been determined (Fig. 3), have also been identified by our sequence analysis.

Our data show that the IBU-degradative phenotype of the strain MPO218, as well as that of the 34ibu and 38ibu consortia, is eventually lost in an interval of 20–70 generations when selective pressure is relieved, and this may probably also be due to recombination between copies of IS*6100* (our data and [26]). Alternatively, the absence of selective pressure could also cause the loss of some component of the consortium necessary to grow using IBU as carbon source. The involvement of this ISs in the generation of these DNA rearrangements, loss of DNA fragments and degradative phenotype has also been proposed for other pathways in sphingomonads, such as a *lin* genes for lindane degradation located in a plasmid [101]. Nevertheless, the IBU degradation phenotype should be stable enough to persist under the WWTPs environmental conditions, as they have been found in all distant locations (Seville, Úbeda and Barcelona in Spain, or Hamburg in Germany). These results suggest that IBU, together with other compounds with similar structure that are degraded in WWTPs using these genes, must be in sufficiently high concentrations in this environment to select for their presence and maintenance. As an example, it has been reported that the concentration of IBU in the WWTP influent from which MPO984 was obtained range from 16.10 to 231.83 μg L^−1^ [102]. The stability of these sequences in species growing in WWTP is a feature that will facilitate future designs of IBU biodegradation systems using bacterial strains.

In addition to the mobilisation of the coding sequences of the genes involved in IBU degradation, we have detected an unexpected event of relevant importance, which refers to the evolution of the regulation of gene expression of these gene clusters. In *R. wittichii* MPO218, the upper pathway was found to be constitutive ([26] and Fig. 4). However, in all the consortia analysed, we found a clear induction of this pathway, which is very evident in the MPO984 consortium, which uses the IBU very efficiently, but even more so in the consortia obtained from MPO977, 34ibu and 38ibu, which show a much more modest induction. We therefore propose that the evolution of consortia is not only a change in species composition or a turnover of genes within or between species, but that there also appears to be an evolutionary adaptation in the expression of the rearranged genes, adjusting them to more favourable situations for induction of expression in the presence of the molecule. This may seem inconsistent given that in general, the elements of specific regulation by the compound to be degraded are often located close to the catalytic genes [103–105] and genomic rearrangement caused by transfer to a new host could more likely lead to uncoupling of these sequences, and loss of regulatory capacity. In the case of MPO218, it could be that it has recently received the *ipf* genes and does not possess the regulatory elements of the upper pathway, or that in the new consortia, there is a transcriptional regulator (in another microorganism) that has changed substrate specificity and is now able to induce the upper pathway expression efficiently. The genes involved in the IBU degradation pathway should have adapted to carry out a process for which they were not originally designed, given that IBU is a compound that has only recently been incorporated into the environment. The identification of a regulator that has accommodated a possible recognition of a novel inducing molecule within the universe of metagenomes comprised in the consortium makes it very interesting as a model of regulatory evolution and adaptation to new contaminants. The identification of this mechanism remains as an open question for the future and provides a new opportunity to explore the evolution or adaptation of biological processes to new evolutionary constraints or to the appearance of new emerging pollutants in nature.

Our findings offer valuable insights into the intricate adaptive evolution that enabled organisms to thrive in a low-nutrient environment. This involved rearrangements of metabolic genes, which led to varying transcriptional outcomes. These results highlight the importance of investigating functions rather than just taxa, as it enables the identification of pivotal events that alter community composition under changing conditions. Additionally, our findings could aid in comprehending the process of biodegradation and stimulate the development of biotechnological solutions, not only for the remediation of IBU but also for other pollutants posing a threat to the biosphere.

## Supplementary Material

Figure_S1_wraf014

Figure_S2_wraf014

Figure_S3_wraf014

Figure_S4_wraf014

Table_S1_v8_wraf014

Table_S2_v8_wraf014

Table_S3_v8_wraf014

Table_S4_v8_wraf014

Table_S5b_v8_wraf014

Suplementary_file_S1_wraf014

Supporting_information_wraf014

## Data Availability

All raw sequences obtained through metagenomic sequencing are available at the NCBI Sequence Read Archive (SRA) database under the Bioproject PRJNA1061829. The 16S rRNA gene amplicon sequencing data can be accessed under the Bioproject PRJNA1209662. The assembled contigs and all the binned genomes recovered from the metagenomic assemblies are available in Zenodo (https://doi.org/10.5281/zenodo.14220178). The plasmid sequences mentioned in the manuscript can also be found under that link. The circular plasmids, contig_127 (named pIBU219), and contig_218 (named pIBU220) are available in the NCBI under the following Genbank accession numbers, respectively: PQ650603, PQ650604.
