# Supplementary figures and images for: Genetic and species rearrangements in microbial consortia impact biodegradation potential
Source: ISME J. 2025 Jan 25;19(1):wraf014. doi: 10.1093/ismejo/wraf014 (PMC11892951; doi:10.1093/ismejo/wraf014)

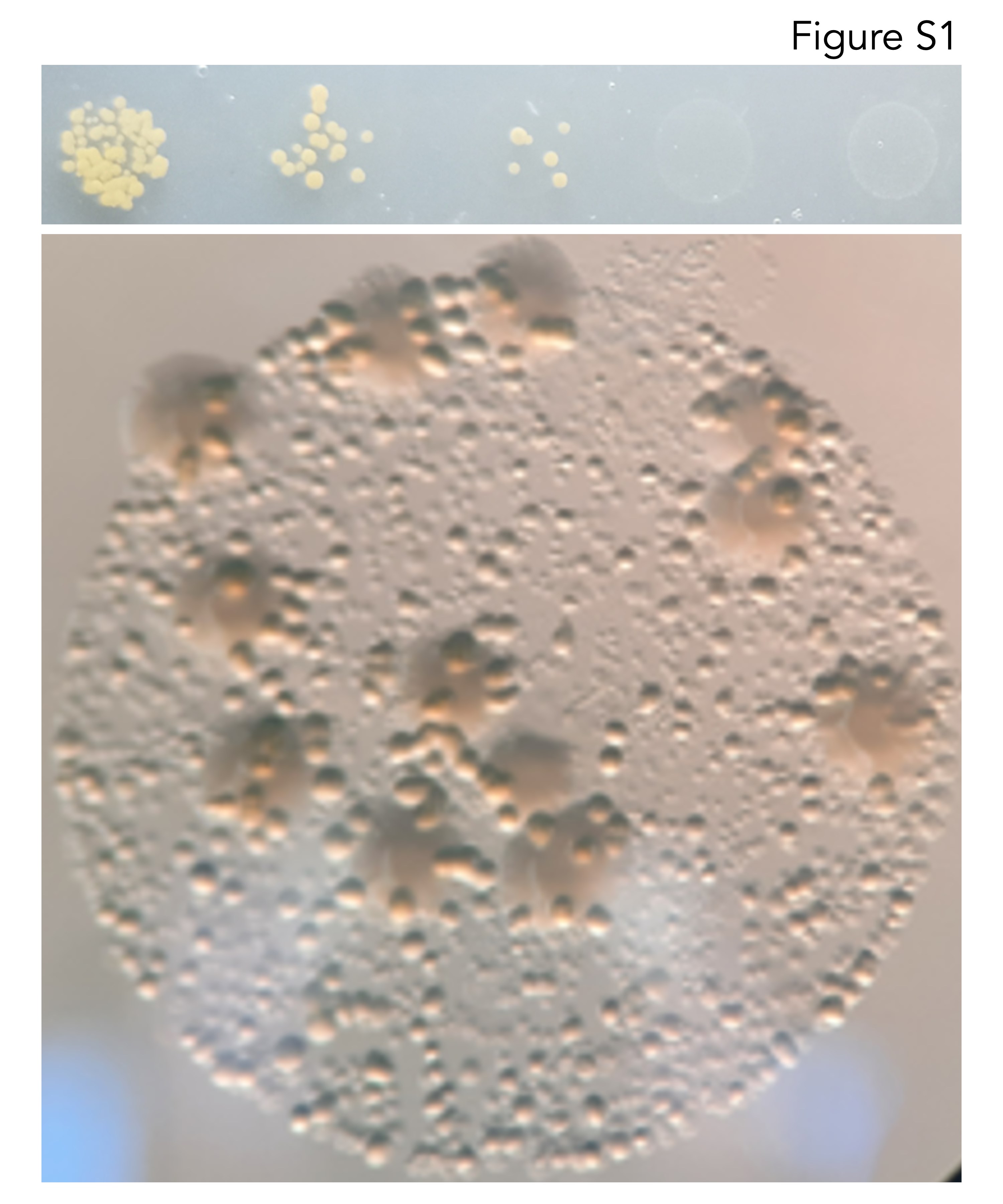

Supplement: Figure_S1_wraf014 [file figure_s1_wraf014.jpeg]

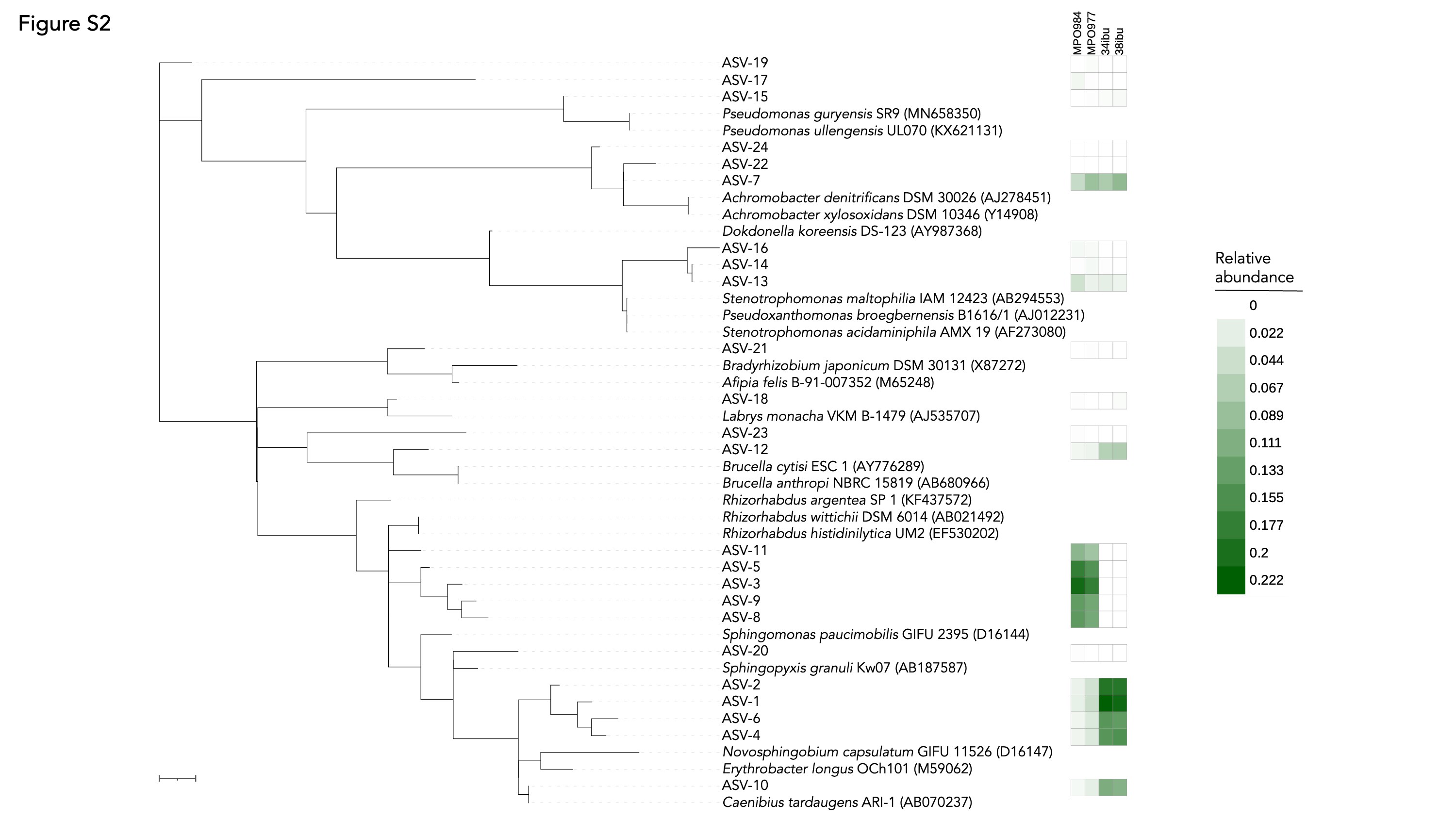

Supplement: Figure_S2_wraf014 [file figure_s2_wraf014.jpeg]

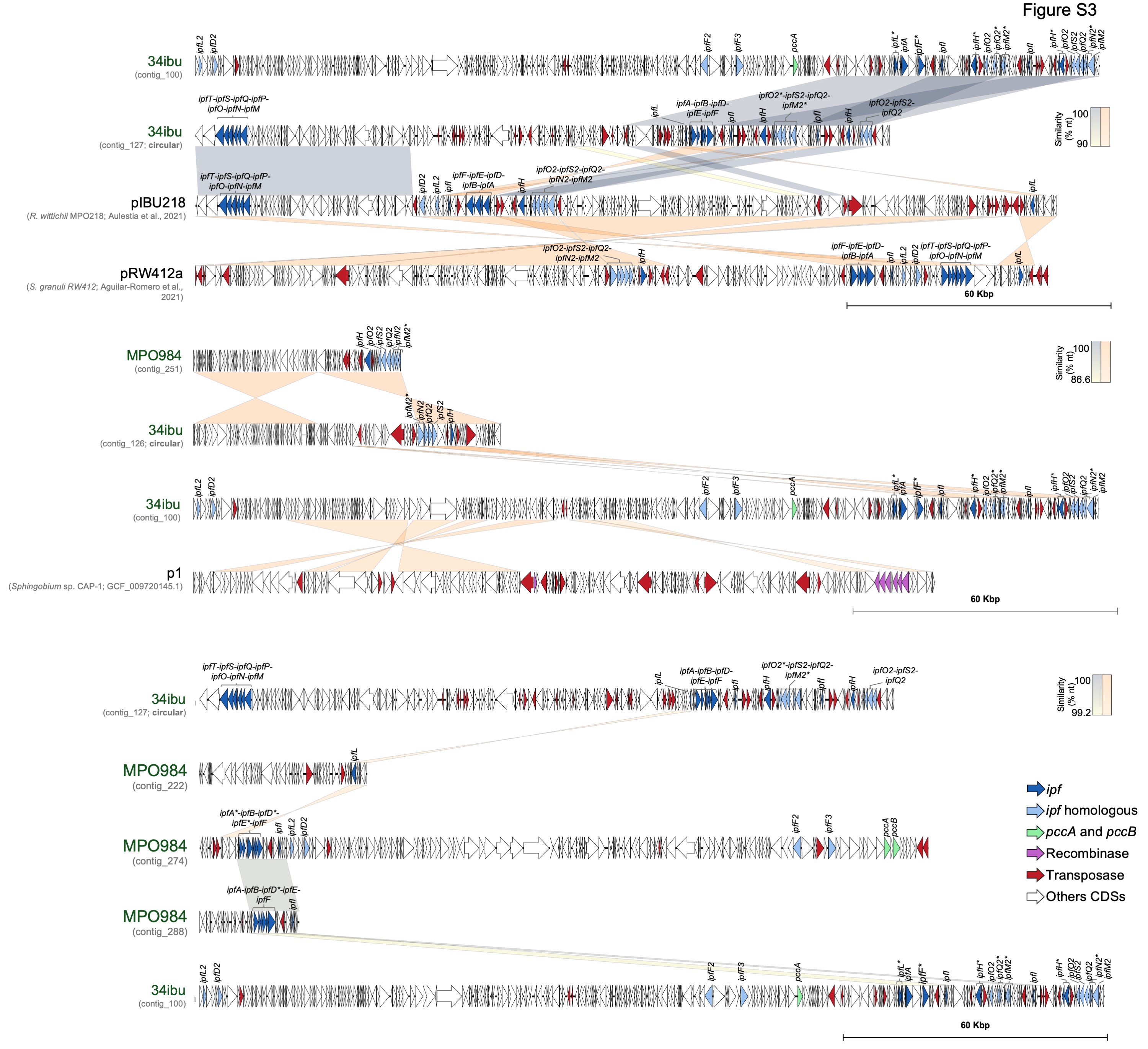

Supplement: Figure_S3_wraf014 [file figure_s3_wraf014.jpeg]

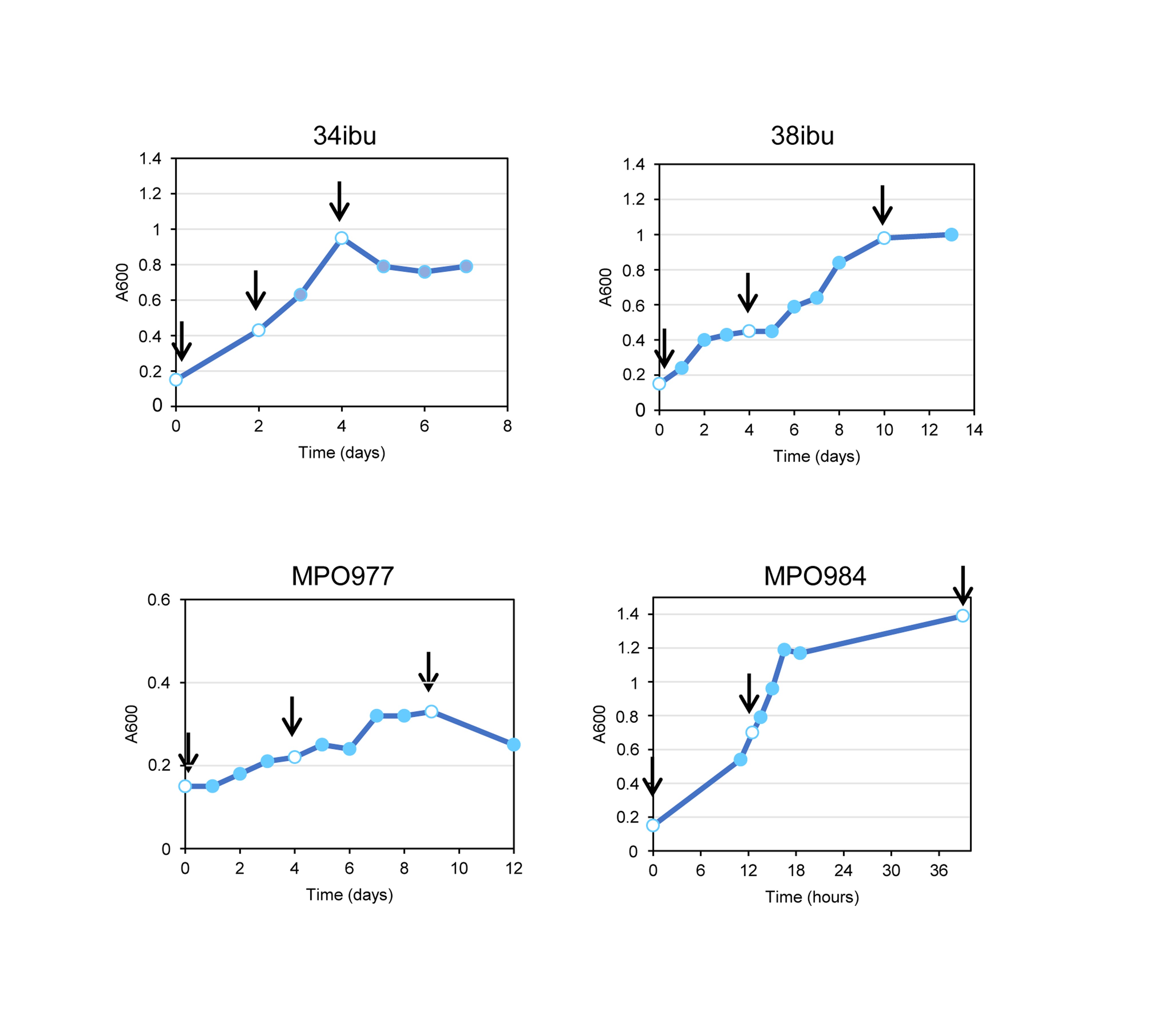

Supplement: Figure_S4_wraf014 [file figure_s4_wraf014.jpeg]
