# Supplementary material for: Genetic and species rearrangements in microbial consortia impact biodegradation potential
Source: ISME J. 2025 Jan 25;19(1):wraf014. doi: 10.1093/ismejo/wraf014 (PMC11892951; doi:10.1093/ismejo/wraf014)
Supplement: Table_S5b_v8_wraf014 [file table_s5b_v8_wraf014.docx]

**Supplementary Table S5.**  Oligonucleotides used in this work.

| Oligonucleotide name | Sequence (5´-3´) |
| --- | --- |
| IpfA-Fw | AACAAGCTGACCCACACAGG |
| IpfA-Rv | CGATGCGCGCAACGGTTGG |
| q-IpfA-Fw** | GGCAAGTACACAAACGATCAC |
| q-IpfA-Rv** | TACGGTCTCGCTCTATCAAG |
| q-CDO-Fw | TGTGCGGGTTCTCGATATCG |
| q-CDO-Rv | CGCCTTGTAGTAGGTTTTGC |
| q-ipfF-Fw* | AGATGGATCAACGCTCCG |
| q-ipfF-Rv* | GCTCATACACCTCGATGC |
| q-ipfF-newF*** | CAAGAGACCTGGTTAAGC |
| q-ipfF-newR*** | GAGCGTTGATCCATCTCG |
| q-ipfI-Fw* | GAGTGGATGAGGTGAAGG |
| q-ipfI-Rv* | TCGGTCAGCATCGCATTG |
| q-ipfI-newF*** | TTGCGGTCTATGATGTCG |
| q-ipfI-newR*** | GTGCGTGCAGATGTTGTC |
| q-ipfM-Fw | GATAGCGAGGAGGAAGTG |
| q-ipfM-Rv | AGATCACGCAGGAACCAC |
| q-ipfM-Fw2** | CCTCGATAAGCGGCAAGA |
| q-HMDH-Rv** | ACGCCCACCCTCGTTCAC |
| q-IpfQ-Fw | GATACCGTCTATTGCGAG |
| q-IpfQ-Rv | AGGAAGATCGACACCTTG |
| q-IpfQ2-Fw** | CGATACCGTCTAATTGCGA |
| q-IpfQ2-Rv** | TGCACTTCGGGCGATCATG |

*These primers were used to amplify the *ipfF* and *ipfI* genes in the 34ibu, 38ibu, MPO977 and MPO984 consortia.

**These primers were used to amplify the *ipfA, ipfM* and *ipfQ* genes in the MPO984 consortia.

***These primers were used to amplify the *ipfF* and *ipfI* genes in *R. wittichii* MPO218.
