# Supplementary material for: Genetic and species rearrangements in microbial consortia impact biodegradation potential
Source: ISME J. 2025 Jan 25;19(1):wraf014. doi: 10.1093/ismejo/wraf014 (PMC11892951; doi:10.1093/ismejo/wraf014)
Supplement: Suplementary_file_S1_wraf014 [file suplementary_file_s1_wraf014.docx]

>IpfF IpfF

MLARDLVKRCARNYPTKTAYLCGERSRSWREMDQRSDRFGVALQQLGHRPGEAVAILTQESIEVYEHFFACMKIAAPRVGLNTGYVWPEMLHVLKDSEVKFLLLDTRCRHLLAERLGELKALGITLIGYGAGHGLERDYESLLATAEGEPHWPALAPDDILFVSYTSGTTGVPKGVMLTQEGGVNCILHSLISFGFGPDDVWYMPAASAWVVVILNAFGLGNGMTTVIPDGGYQLQAYLRDIERFRVTVGMLVPTMLQRAIVEIQTNPVYDLSSLRMVVYGSSPATPKLIRDARATFKGIKLLQAYAMTEATGGWISYLTDADHEHALREEIELLKSVGRIGIHYDCSIRDESGQPVPIGQSGEIWLRGNTMMKGYRNLPEATAEAMPDGWLRTNDIGRLDERGYLYLLDRQKFLIITGAVNVFPTTVEAILVEHPAVEEVAVVGVPHPEWGEAVVAVVVRKPSHRDVTVQALIDFCHGKLSRPETPKHVVFVDELPKTSNAKLKKGELKKWLSGGAVPLPWQLEVA

>IpfF2 IpfF-contig_100_pilon-1

MVSKIAIDAAASETASNDIAPEQRSYEARRVAYSPQIPAEFKATDIVDEWASSAPEALALISLNAAGELVDTRTAAELVADSCAVAAGLLKLGIKKGERIFIMLPRVSAWYAAMLGAMRIGAVPMPGPNMLTAKDIGYRIAKGRAVAAITDLAGAAKLDAAPLGEDTLRHRIAWRGDATDVPAGWLDFADLLATADLPASFEPLAATDPLLIYFTSGTVSHPKMVEHAQSYALGHVATARFWHDLGPGDRHWTVSDTGWAKAAWGGLFGQWHERATVVQVALAKPDTNLILGIIARHKITSFCAPPTLYRQLILGDFSPFDLSALKHCTSAGEPLNPEVIRVWEEKTGLRIYDGYGQTETTVLVANFPGLPIRPGSMGKPVPGYDVDIRDEDGSRIADGVAGAIAVRTEPHPVGLFQGYFEDPDATASRFQNGWYFTGDRGVRDPDGYIWFEGRDDDVITSSAYRIGPFEVESALLEHPAVAEAGVVGRPDPLRTEIVCAFVILTPGHEGSEALTAELQDHVKRTTAPYKYPREIRYVSTLPKTISGKIRRTELRAELIAEMAAEKKETE

>IpfF3 IpfF-contig_100_pilon-2

MNLSWWLERASAEYPQKTAIVDASGASITYAELEALVSQIGHVLRDDMGVRPDDVVVTCMADNYLHVAIMYATMRIGAIFSGLNHKMVHEKFHSDIDRCRPKAAIVAPEFPEIADLISGYPEIAVAMTTGGYGGLPNLDSMAAGKTGLLKVEPRSRDDIAAINFTAGTSGASKGVIFTHGKLETSCWGSIFLAGVKSDCRNLSLVGMFHSGGIADAVRLVMVGGTLLWSEGWDVDRVVNIIKTYKPNFAYYIVPTMMRDLMRHPEWEDLDIHGLRTHVSGEVVPPEIEAAMRAKGAIVGAMYGMTETMPVRALSSALVYRDEDELPRGSSGRPNKEFCEVVLKDPHSGEILEGGDVEGEICLRGDVVTPGYYKDPERTAGAFDEDGYLHTRDRGYRDADGWYFIRGRTDDMILSGAEKLSLLEVDKVLLEHADVRDAACVGVAHERFGEVPAAFIVLSADHSESAARDILDSYCISAMERWKRPRLYIFVDEIPRTAAKQTKMSGELRRIVADITVANADGVVTLGELQARGIVAAAANAG

>IpfA IpfA

MASTQTITITRKAPDVDVGALIERDRIHGSLYANESIFELEMKKIFYDGWVFVGHDSEVPTAGEYVRRTLGREEVLMVRQRDSSIAVIANRCAHRGNMMCIANHGKEKYFTCTYHGWVYDLAGNLKDVPYPGGFDKDKSELKLQPLRTEVYRGFVFATFNASAPPLMEQLGRGKILIDRACDMSPTGRLQLTAGWTKQRFGANWKMLPENDTDGYHVNDVHASFAQVIDSHYDSAAIAAEDSLRSQAKDWGNGHTELYLSPTYTEYLKWFNTTPNRFPEYIAQMKAAYGEEKGDNILRDGPPHATIFPNLFLGEMNIIIFLPINAHECVQWHTPMLLEGAPDEVNQRIIRNSEAAMGPSAFLLADDSVISERQQIALRDRADWLDVSRGLNREHVDEMGVVVGHVTDECTNRGFWQHYKKVMTAPSPSPV

>IpfB IpfB

MTSITAAVGTKQKTDVIGLEEHREVCDFLYREARLADESRYAEWEALVEDDMTYWVPRGEGDYDMNKHVSITADNRSRLRVRIAQLMTGKRHAQLPVSSMRRIVSNIEVEHHAQGGYRVLSNFVLYELRRSSTGQIEVWPGRVEHHLRRRADGSLGMFFKKVVLIHGDEAVPSLAFII

>IpfH IpfH

MIASVVIVGANLAGGRAAEALRLNGYEGRIVLIGEERWLPYERPPLSKECLWDRDQLPENFFLHDQQWYEDNKIELELGVRAEALELSGRGVRLASGKEIPADRILLATGGKARLLPLDGATAANVHHLRTKDDADRLAADLKPGARIVVIGMGVIGAEVAASARKSGCEVTAIEPAPVPMIRTLGAHFGAWLGREHDKRGVKARYGIGVTRLYLDGGLVRTVELDDGTRIDCDAVVVGIGIVPSTELAANAGLAIGNGIVVDRQGRTSHEAVFAAGDVADQPNFFGGRVRLETYQNAADQGMAAAQAMIGREVDYLKPCWFWSDQYDINIQVSGRIDDSLPVVMRGELDSSQFTAFFLDGNVVAGVLTANRAVDMGVGKRMVERRLEVDPGQLGDASIPLREFLKPKARAA

>IpfI IpfI

MSELIRLCRVDEVKEGEPVAAHVAGLPPFAVYDVGGTYYVTDNICTHGNAMLTDGYQDGGTIECPFHGGAFDIASGAATVFPCQIPLKTYSVEVDDGWIAIRLASPEAGA

>IpfD IpfD

MVTRVKKKTRARAAIAGLGFSAMSRQPVGTIRELAATAVAAAAADAGLRLQDIDGLLLNKSPAAEPEELPLRLQNDLGLRDLGLLAAMDSEGSTAVQMVQYAAMAVREGLVKSVACVFADTPLKGSGAGGGDAFALAMPLTGVEGWEAQQGFLGATAAYALAARRHMALYGSTAEQLGAYALACRQWAALNPQAFLRKPMTMDDYLASPFVVEPFRVFDCCFPVNGAVALIVTSADRAVDGPQPPVFIHGMGQGHRGRSGLSGDDPEVFTGAIQAGQTAYRSAGVNASDVTQCQFYDAFSYAGILGLEAYGLCPRGEGGAFVAQGHTAPGGKLPVNTGGGHLSGFYLQGMTPLSEAVIQARGAGGARQVVRNDLILVTGNGGCLDYHTCVLVSPHRTLA

>IpfD2 IpfD2-homologue

MLSDKTAIAGIGATEFSKNSGRSELRLASEAVCSALADAGIEASQVDGLCTMGADNTSEVEIARAIGAGELTFFSRIPFGGGGACAVVQQAALAVASGLASTVVCYRAMNERSQYRFGGPLPSAPTAEGEVTHYHTLHGLATAASFVAVMIRRYMHQYGATRQDFANVAIAARKHAAVNPAAFFYGKELTLDDYLGSRTISDPLHLYDCCMESDGAVALVVTSAERARDLKTKPVMVRAAAQGAARGNIPLMGFYGNDIIPFEDTRIVSEQLYAMGGLSAKDMDAAIIYDHFGPTILPALEASGFCNRGEAKDFIRDGNIEIGGGLPVNTHGGQVGEAYIHGMNGIAEAVRQVRGSAINQVVGLENILVTSGGGVPTSGLILGN

>IpfE IpfE

MDSTFFHRYDEAFLAFIAAGELRIPVHTETGRALGLHQRAWCVAGDHGVQWRPASGRGAVLSFTVTRRPYTPEFPVPLVHGLIELAEGPRLICRLDGVTPEAVAVGQAVQAHFDRQGLVFRPALDDGNKADK

>IpfL IpfL

MSIHGVLRLGEICVRVLDIEASRRHYGDYLGLIETGRDDDGKTYYKAWDEHDCHSVVIRPADHCGVDYVAFKVLDDDALTELDRRLQDYGVETRQIPSGTYARSGRRVEFTLPTGHVCQLYAFKEQVGNGMDAANPGTIPDDGYIKGMRIIRLDHCLLGGRDIAASRDLFIKVFGFNLTEELQDHESRAALATFVSCSNKPHDIAFVAQPEDGKFHHMSFLLESVSDLYHAADRMGKYDIPIDFGPNRHGVTRGATVYFFDPSGNRNEVFTGGYVYYPDNPALIWDTSKFGNALFSQGNVVVPSFLNVVS

>ipfL2 ipfL2-homologue

MAVTGVLRPGYVQMRVLDMEQAIPHYRDRVGLDQVGETEGDRAFFRGFDEFDRHSIILREADTPGLDVIGFKVASDRDLDVFKKRIDAMGVRTEEIPAGADPGVGRKIRFTTPTKHVFDLYADMEISDTAPMTKNPDVWHREPRGMRVQRFDHCALNGTDIVGSTKIFVEALDFEVSETVVDESNGTELAVFMSCSNKAVEDLPKGVTGKIDKFALRQEFRNNLDRLPWTV

>IpfL3 IpfL-contig_208_205_pilon

MVDESNGTELAVFMSCSNKAHDIAFIGFPEDGKLHHTSFRLESWNDVGHAADIITRYDISLDIGPTRHGITRGQTIYFFDPSGNRNETFSGGYDYYPDNPRRVWSADEAGKAIFYYERQLNDRFMAVNT

>IpfM IpfM

MNDPVATSGARGALPEIRHFIDGDYRASISGKTFPKHRPYDGRHILDVNEGGREEVDAAVSAARTALQGPWATISVDDRADLMRAIADGITKRFDDFVSAEMNDTGQPISAMRHAFVPRGAANFNAFADLVKSVSTECFHTETPDGRGALNYAIRKPKGVIGVISPWNAPFLLMTWKVAPALACGNTVVVKPSEETPSTATLLGEVMNEVGVPKGVYNVVHGFGPDSAGAYLTEHQGVDAITFTGETRTGTAIMQAAARGVRDVSFELGGKNSAIIFADADLDAALEGLMRSVFLNTGQVCLGTERVFVERPLFERFVGALGERVKSLKPGHPEGAGTSFGPLISSEHREKVLGYYRRARADGAVIVAGGGVPELPAELSGGSWVEPTIWTGLPDDHPVIEEEIFGPCCHVRPFDSEEEVIGLANNTSYGLSSAIWTQNLSRAHRMAAAIDVGITWVNSWFLRDLRTPFGGTRQSGIGREGGVHSLEFYSELRNVCVKL

>ipfM2 ipfM2-homologue

MARGDEPLPTVTAEVLNYIDGAYRKGSEGKSFVNIDPATGKEIGRVYEASREDVDAAVQAAKRALKGPWGKMTIPERVKLIIKVADEIERRFDDFLAAEVADTGKPISIASHIDIPRGAANFRMFADVVSTTPTESFMTPTPDGSKALNYAVRKPKGVVAVVCPWNFPLLLMTWKVGPALACGNTVVVKPSEETPRTAALLGEVMDAVGMPKGVFNVVHGFGPDSAGEFLTSHKDVDAITFTGETGTGTAIMKQAAVGLRDISFELGGKNPAIVFADCDIDKAIEGVARATFLNTGQVCLGTERIFVERSIFDKFVERLAAAAQAFEPGDPKHPQYMGPLISDEHRSKVLGYYQRAVDEGATVVTGGGVPKNVDGGFYVEPTIWTGLAPDSTVMREEVFGPCCGVIPFDTEEEAIAMANDTPYGLSATIWTQDLSRAHRVSAEMEVGICWVNCWFLRDLRTAFGGAGQSGIGREGGVHSLEFYTEIENICVKL

>IpfP IpfP

MPIISVTIAEGRQVEKRRTLIKALTEATVNAFDVRPDQVRVILNEVPLENYAVAGVTFAEQALLPGASK

>IpfO IpfO

MLETATIHAIADIVVGAQDGAYTITKFTDAHPDMTIEDSYAVQDELLRRWQARGRKLVGLKAGLTSKAKMDQMGVHVPSFGILMNDTLDPDMGVVPMDKLIHPRVEAEIAFVMKDALSGPDVSVDEIITATDFVQPAVEIIDSRFEKFKFDLVSVVADNGSSARFVMGGRARRPQELSLDTIGIVFEKNGEVVATASSAAVLGHPARAIQMLVAWLHERGRVLPAGTIVLTGGATEAVAVAPGDFISARFQDLGAVSFRMGQKDTE

>ipfO2 ipfO-contig_251_pilon

MGLTSRAKMQQVGVDEVAWGRLTDAMLLEEGAALSLSRFVHPRIEPEIAFLMKAPLAGKVTAAQALACVEAVAPAMEVIDSRFENFKFALVDVVADNTSSSGLVVGGWGDPMQDLSNLGVILEINGEVVEVGSTAAILGHPLRSLVAAARLIGGEAGETINAGDIVMAGGITAAPTLKAGQTIRNTVQNLGSVSITVEA

>ipfO2 ipfO2-homologue

MTSLAEYAEILDRAAHEAHATPQITHSNDKLTVADAYAIQKLSVERRLARGEKRIGVKMGLTSRAKMQQVGVDEVAWGRLTDAMLLEEGAALSLSRFVHPRIEPEIAFLMKAPLAGKVTAAQALACVEAVAPAMEVIDSRFENFKFALVDVVADNTSSSGLVVGGWGDPMQDLSNLGVILEINGEVVEVGSTAAILGHPLRSLVAAARLIGEAGETINAGDIVMAGGITAAPTLKAGQTIRNTVQNLGSVSITVEA

>IpfN IpfN

MALETNTIERLGDELFEALAANRTVPNLRDRNPGMDIVDGYRVQERMIARRLAAGESIVGKKIGVTSKAVQQAIGVFEPDFGQLTSAMVVEDGAAIDLDRLIQPQAEGEIAFVLKQDLIGPGVTATDVLRATDYVAACFEIVDSRIDDWDIRIQDTVADNASCGIYALGRERFDPRDVDLTLAGMVLERNGELAATGVGAAVQGSPLNSVAWLANTLGRLGIPFRDGEAILSGSLGPMIRIADGDRLSVRIGGLGSCSLSFARGGRNA

>ipfN2 ipfN2-homologue

MADQEVTFDRSNIDKAAAQLRHAAETMNPGPPIRDLISAGGVEAAYAVQDANTTHYLANGRRLVGRKTGLTSKSVQKQLGVNQPDYGMLYADMDVPEGQPILLTRVVQPKVEAEIALVIGRDLDDPDVTTAEMLRAIEYVVPAIEIVDSRIANWDIKIWDTIADNASSGVYTLGAVPKKLDGLDLRTCGMVMERRGDPISVGAGVACLGSPISAALWLAQVMAKAGRPLKAGDVILSGALGPMAGVAAGDVVEARINGVGTVKAFFEAGA

>IpfS IpfS

MTLEAQVRGRKVKMHDMCLRDGMHSMRHQITIEQMIDIATGLDEAGVPLLQVTHGDGLGGSSLNYGRGLHTDKEYITAVASRMKNARVSVLHVPGIGTLDELRMAADCGAKCVHVASHCTEADVTEQHIGLGLKLGMDTVGFLMMAHMTPTANMVEQGLLMESYGAQTVYCVDSAGYMLPDDVTDRIASLRAALKPETEIGFHGHHNLGMGVANSLAAVAAGADRIDGSAAGLGAGAGNTPLELFAAVCDRLGVETGVDTFKLMDVAEDRVLPIMPRPPRADRDSITLGYAGVYSTFLYQAKDAHRKYGISARDLLLELARKKMIGGQEDMIEDTALTMAKEQGLIPA

>ipfS2 ipfS2-homologue

MTLYPTQTKLYIQDVTLRDGMHAILHNYGTESVRTIAKALDDAGVDAIEVSHGDGLNGSSFNYGFGAHTDWEWIEAAADVIEKAVLTTLLVPGIGTVEELKRAYALGVRSVRVATHCTEADVGKQHIGIARDLGMDVSGFLMMSHMLEPEALAQQALLMESYGAHCVYVTDSGGALDMDGVRARLQAYDRVLKPETQRGMHAHHNLSLGVANSIVAAQEGAIRIDASLAGMGAGAGNAPLEVFIAAIDRKGWKHGCDVMALMDAAEDLVRPLQDRPVRVDRETLSLGYAGVYSSFLRHAEKAAEQYGLDTREILVELGRRRMVGGQEDMIVDVALDILSARKGETEPKGEMAA

>IpfQ IpfQ

MTKIRCAIIGPGNIGTDLLYKLLRSEVLEPVWMVGVDPTSDGLARAKELGLRTTADGVDGLLPHVVEDRIQIAFDATSAYVHPENSRKLNALGVLMIDLTPAAVGPLCVPPVNLATLADQAVMNVNMVTCAGQATIPMVNAVSRVQPVDYAEIVATVASKSIGPGTRKNIDEFTRTTSGAVARVGGAKVGKAIVVVNPAEPPLMMRDTVYCETAEDPDQEAITRSVRDMIAEVQKYVPGYRLKNGPTFEGRKVSIFLEVEGLGDYLPKYAGNLDIMTAAGARTAEMFAREILDGTLELKPIVVEAA

>ipfQ2 ipfQ2-homologue

MSKIKCAIIGSGNIGTDLMIKILKGSDSLELAVVVGIDEKSEGLAMARERGVATTHDGIEGLKKMGSYPEIGVVFDATSAYAHKVHDEILRADGKQIVDLTPAALGPFTVPPVNADSNLDAVNVNMVTCGGQATIPIVAAVARVTKVHYAEIVASVSSRSAGPGTRANIDEFTRTTAKGIEVVGGAEKGRAIIILNPAEPPMIMRDTIFTLTDQVDEDKIRQSILDMIATVQSYVPGYRLKQEVQFERFGSNNPVKIPGYGEFAGLKTSVFLEVEGAGDYLPKYAGNLDIMTAAAKAAGEMLAARMLNRKAAA

>IpfT IpfT

MDFFPPVAEQIFALEHAAGLARIAEHADFAEATRETVEAVLQGIGVLAAGEYAPTNRVGDTAGPRWIDGRVEMPAAFHTAYRAFVEGGWGGIAVPTEHGGMGLPFSLAVAAMESLGTANMGFGLIHLLSFGAIHAIEVYGSDHQKAVWLPHLVNGRWNGTMNLTEPLAGSDVGALRTRAEQAADEGLWRIRGQKIFITFGECDLVENVVHLVLARTNDAPKGTKGISLFLVPKLRLDDQGRPAIPNGVHCVSIEHKLGIHGSPTCVMAYGEDGEECLGELVGEIGGGMRAMFVMMNRARLLVGNQGVQIAERATQQALAYAHQRIQSARASDPVSGAVAIIEHPDVRRMLLRMKSLTQAARALTYACAGAIDMGAAGDDKAAARAEVLTPLAKSWASDIGCEVASLGIQVHGGMGYIEETGAAQHYRDARIAPIYEGTNGIQAADLVGRKLAIADGAAIGDLIEDILAECGDEPRLDALARACLEVLAWLKDRPVDDRLAGSHDFLTMLAVATAGWLMLRQARAAEAGLAGDADNAWLQAKAATTRFFLDRIVPEALGRKAGAIAGSEGLYSVSTKALIGVQ

>PccA PccA

MFKKILIANRGEIACRVIKTARRMGIKTVAVYSDADARAPHVEMADEAVHIGASPASESYLIADRIIAACQATGAEAVHPGYGFLSERTSFAEALAAANIAFIGPPANAIAAMGDKIESKKLAMEAGVNVVPGYVGEIDDTDHAVRIANDIGYPVMMKASAGGGGKGMRLAYSEKDVRENFDSVKREGLNSFGDDRVFIEKFIESPRHIEIQVLGDKHGNIVYLNERECSIQRRHQKVVEEAPSPFVSPEMRKAMGEQCVALARAVGYFSAGTVELIVSGADKTGQGFYFLEMNTRLQVEHPVTECITGLDLVEQMIRVAYGEKLAFTQADVKINGWAVENRVYAEDPYRGFLPSTGRLVRYRPPAEVDNVRVDDGVYEGSEISMFYDPMIAKLITWGETREAAIDRQITALDRFEIDGIGHNVDFLSALMQHPRFRSGNITTGFIAEEFPEGFTGAAADAKLGLHLAAVAGVLASIDTARAAQIDGRLNGPADPSTSWVVTLDKVERQIEILGDAVSVDGTPVVVDAPYVPGQKLVEAVIDGEPLSIRVERKRTGWRFTTRGASHELRVLTPRVASLAHHMIEKIPPDMSRFLLCPMPGLVTAIHVAEGAKVEAGQPLAVVEAMKMENILRAEKAGTVKAVAAKAGDSLAVDAVILEFE

>PccB PccB

MLAILQKLEEKRAAARLGGGEKRIEAQHRKGKLTARERIEVLLDADSFEELDMYVEHNCTDFGMEAEHIPGDGVVTGSGTVNGRLVFVFSQDFTVYGGALSERHAQKICKIMDMAMKVGAPVIGLNDSGGARIQEGVASLGGYAEVFQRNILASGVIPQISVIMGPCAGGAVYSPAMTDFIFMVKDSSFMFVTGPDVVKTVTNEVVTQEELGGAVTHTTKTSVADNAFDNDIEALLSVREFVDFLPLSNRHDLPERPTDDPWDRAEPSLDTLIPDSATKPYDMKELIAKVVDEGDFFEVQPNHAGNIVIGFGRIEGRPVGIVANQPMVLAGCLDINSSKKAARFVRFCDAFDIPIVTFVDVPGFLPGTAQEHNGIIKHGAKLLFAYGEATVPKITVITRKAYGGAYDVMASKHLRGDLNYAWPTAEIAVMGAKGAVEIIFRKDIGDAAKIAERTREYEDRFANPFVAASKGFIDEVIMPHSTRRRIALGLRKLRNKSLENPWKKHDNIPL
