# Supplementary material for: Genetic and species rearrangements in microbial consortia impact biodegradation potential
Source: ISME J. 2025 Jan 25;19(1):wraf014. doi: 10.1093/ismejo/wraf014 (PMC11892951; doi:10.1093/ismejo/wraf014)
Supplement: Supporting_information_wraf014 [file supporting_information_wraf014.docx]

**Supporting information**

**Figure S1. Visualisation of the colonies from consortium 34ibu.** Top: Colonies of consortia 34ibu plated on MM with IBU on serial 10-fold dilutions. Bottom: Close-up image of a 10^7^ dilution showing colonies with different morphologies visualised in a stereomicroscope (Leica S6E, 2x magnification).

**Figure S2**. **Phylogenetic tree of the top 24 amplicon sequence variants (ASVs) in the bacterial consortia**. This tree includes the top 24 16S rRNA gene ASVs identified in the samples analysed in this study. Sequences from closely related type strains were included as references to provide better resolution and clarity regarding the taxonomic placement of the query sequences. The abundance of each ASV in the samples is represented in the heatmap on the right as a fraction of 1. Scale bar = 0.01 substitutions per nucleotide.

**Figure S3.** **Mobilisation of *ipf* genes across different plasmids.** This figure illustrates a selection of plasmid variants from different backbones, each sharing *ipf* loci with high nucleotide similarity (represented by a yellow-to-grey gradient, as indicated in the legend). The nucleotide similarity of inverted regions is indicated in orange gradient. Coding sequences (CDSs) are color-coded based on their functional classification. All *ipf* genes and their homologous sequences are annotated with gene names above their corresponding CDSs. plasmid P1, from *Sphingobium* sp. CAP-1, shares a large DNA region with contig_100 (Backbone-2) despite lacking *ipf* genes. Each replicon is labelled according to the sample from which it was assembled, irrespective of its relative abundance (Figure 2b)**.**

**Figure S4**. **Metabolomic analysis of the consortia MPO984, 34ibu, 38ibu and MPO977.** Growth curve of the samples used for the analysis of the metabolites, and time points (black arrows, i.e. time zero, mid-exponential and stationary phase) where the samples were taken for the metabolite analysis.

**Supplementary File S1:** Protein sequences (FASTA format) used as “trusted proteins” in the prokka annotation. These sequences correspond to Ipf proteins, which Prokka processes with high priority during annotation. Trusted proteins are used as reference markers for predicting gene functions, and their presence ensures that any matching sequences in the input data are flagged and annotated preferentially with the names and functions of the trusted proteins. Similar sequences (orthologs) detected during annotation are labelled as Ipf-2, indicating their evolutionary relationship to the original Ipf proteins.

**Supplementary Table S1.** Amplicon sequence variants (ASVs) detected in the 16S rRNA gene amplicon sequencing data of this work. The abundance of each ASV in each sample is represented as a fraction of 1. The raw taxonomical classification of each ASV is given according to the RDP database. The confidence value reflects the probability assigned by the classifier to the taxonomic assignment, providing a measure of reliability for each classification.

**Supplementary Table S2.** Statistics for the long-reads based metaFlye assembly [48]

**Supplementary Table S3**. DAS-Tool-refined bins obtained from the IBU-degrading consortia. **Ochrobactrum anthropi* is now defined as *Brucella antropi* (<https://lpsn.dsmz.de/species/ochrobactrum-anthropi>).

**Supplementary Table S4**. Single nucleotide polymorphisms (SNPs) found in the *ipf* gene sequences in sample MPO984.

**Supplementary Table S5**. Oligonucleotides used in this work.
